# Synthesis and In Vivo Incorporation of 4‐Difluoromethyl‐L‐Phenylalanine Into Proteins

**DOI:** 10.1002/cbic.70541

**Published:** 2026-09-23

**Authors:** Daniel S. Honeycutt, Jason M. Mrosla, Sarah A. Sexton, Giovanni E. Cavalli, Emmerson G. Bartels, Brian Pallares, William K. McCarthy, Ruojun Wu, Fang Wang, Jacob M. Goldberg

**Affiliations:** ^1^ Department of Chemistry University of Rhode Island Kingston Rhode Island USA; ^2^ Department of Chemistry Colgate University Hamilton New York USA

**Keywords:** amino acids, bioorganic chemistry, difluoromethyl group, fluorine, hydrogen bonds

## Abstract

The difluoromethyl group exhibits unique chemical and nuclear magnetic resonance (NMR) spectroscopic properties. Here, the synthesis and characterization of 4‐difluoromethyl‐L‐phenylalanine is reported. Methods have been developed to incorporate this versatile unnatural amino acid into full‐length green fluorescent protein and carbonic anhydrase, using an orthogonal transfer RNA (tRNA)/synthetase pair with standard genetic code expansion techniques. Taking advantage of the characteristic ^19^F NMR behavior of the difluoromethyl group, the potential application of the difluoromethyl group as a probe in protein NMR studies was explored.

## Graphical abstract

The amino acid 4‐difluoromethyl‐L‐phenylalanine was synthesized and incorporated into full‐length proteins using an orthogonal aminoacyl‐tRNA synthetase (aaRS) with standard genetic code expansion techniques. Subsequent experiments with these proteins show the application of the difluoromethyl group in ^19^F NMR‐based protein structure studies.

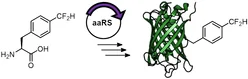

## 1 Introduction

Fluorine is often introduced in small‐molecule therapeutics to modulate biological and medicinal properties [1, 2, 3, 4, 5, 6, 7]. Among various fluorinated functional groups, the difluoromethyl (CF_2_H) group has drawn increasing attention for a number of reasons. First, the CF_2_H group is highly electron‐withdrawing, with a Pauling electronegativity value between that of a hydroxyl group and that of a thiol (Table 1) [8]. As measured by the field‐inductive effect parameter *F*, which is derived from Hammett substituent constants, the CF_2_H group also resembles the thiol and hydroxyl groups [9]. As indicated by Charton parameters, calculated from van der Waals radii, the CF_2_H group is about 20% larger than the thiol group and twice as big as the hydroxyl moiety [10, 11]. In addition to these steric and electronic effects, the high polarity of the carbon–hydrogen bond of the CF_2_H group not only increases the acidity of the CF_2_H group but also allows it to serve as a hydrogen bond donor [12, 13, 14, 15, 16, 17, 18, 19, 20, 21, 22, 23, 24, 25]. Because of these favorable properties, the CF_2_H group has been of synthetic interest [26, 27, 28, 29, 30, 31, 32, 33, 34, 35, 36, 37, 38] and has been employed as a surrogate for hydroxy or thiol groups in new bioactive compounds [4, 39, 40]. Despite the prevalence in synthetic small molecules and peptides [41, 42, 43, 44], the incorporation of the CF_2_H group into proteins is still underexplored, with only a few reports, including difluoromethyl‐containing methionine [45] and isoleucine [46] derivatives. Here, we demonstrate the incorporation of 4‐difluoromethyl‐L‐phenylalanine into proteins via genetic code expansion. Applications of this fluorinated amino acid as a probe in protein ^19^F nuclear magnetic resonance (NMR) spectroscopy are also described.

**TABLE 1:** Comparison of properties of the difluoromethyl group and other moieties.

Substituent	Electronegativity *χ*_*P*_ a	*F* b	*R* c	Charton steric parameter, *ν*	*Space‐filling model*
─CF_3_	3.46	0.38	0.16	0.91	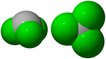
─CF_2_H	3.00	0.29	0.03	0.68	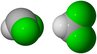
─SH	2.32	0.30	−0.15	0.60	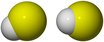
─OH	3.51	0.33	−0.70	0.32	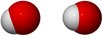
─CH_3_	2.27	0.01	−0.18	0.52	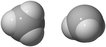
─F	3.98	0.35	−0.02	0.27	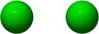

a Electronegativity on the Pauling scale.

b Field/inductive effects derived from Hammett substituent constants.

c Resonance effects derived from Hammett substituent constants.

## 2 Results and Discussion

**SCHEME 1:**
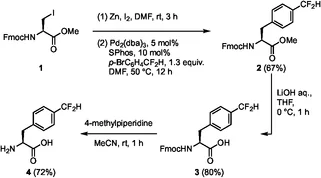
Synthesis of 4‐difluoromethyl‐L‐phenylalanine (dfmF).

To incorporate the difluoromethyl group into proteins, a synthetic route to 4‐difluoromethyl‐L‐phenylalanine (**4**,dfmF) was developed (Scheme 1). Starting with *N*‐Fmoc‐3‐iodo‐L‐alanine methyl ester (**1**) [47], the 4‐difluoromethylphenyl motif was introduced via a Negishi coupling reaction [47, 48, 49, 50] with the Pd_2_(dba)_3_‐SPhos catalytic system [51, 52, 53]. Although an *N*‐Boc protection strategy could be employed, the *N*‐Fmoc protecting group was chosen given its widespread use in modern solid‐phase peptide synthesis, unlocking the potential applications of **4** beyond protein chemistry. The resulting product (**2**) was subjected to ester hydrolysis with aqueous lithium hydroxide. Fmoc deprotection was conducted under standard conditions, allowing for isolation of dfmF (**4**) by simple filtration.

Subsequently, dfmF (**4**) was incorporated into model proteins using a permissive orthogonal tRNA/synthetase pair derived from an *M. jannaschii* tyrosyl‐tRNA synthetase/tRNA_CUA_ system engineered by Mehl and colleagues for the incorporation of 4‐trifluoromethyl‐L‐phenylalanine (tfmF) [54]. Given the permissivity of the synthetase and the observation that the difluoromethyl and trifluoromethyl groups are sterically and electronically similar (Table 1), we hypothesized that the mutant synthetase would be able to incorporate dfmF.

The amino acid was first introduced into a super‐folder GFP (sfGFP) variant with a C‐terminal histidine tag [55]. During the original development of this synthetase [54], asparagine‐150 in GFP was chosen for substitution with a variety of phenylalanine derivatives for two reasons. First, mutations at this position are generally well tolerated. Second, truncation at this position does not produce fluorescence. Accordingly, our proof‐of‐principle studies also began with this construct for the same reasons: the incorporation of dfmF is expected to be tolerated in this position, given the literature precedent for the incorporation of tfmF here and the fact that the resulting fluorescence provides an easy readout of experimental success. The incorporation began by expressing wild‐type sfGFP, which contains an asparagine at position 150, in a pBAD vector system under the control of an auto‐inducible araBAD promoter in *E. coli* cells [55, 56]. The sfGFP was isolated from the fluorescent green culture by immobilized metal ion affinity chromatography and analyzed by gel electrophoresis (Figure S3), intact protein mass spectrometry (Figure S9), and MALDI‐MS of trypsin digests (Figure S12). To incorporate unnatural amino acids [57, 58, 59, 60, 61, 62, 63, 64], electrocompetent *E. coli* cells were cotransformed with a pDule‐tfmF A65V S158A plasmid encoding the orthogonal synthetase/tRNA pair and a pBAD plasmid encoding the same sfGFP sequence with an amber stop codon at position 150. These cells were cultured in arabinose‐containing autoinduction medium under different conditions: in the presence of tfmF, or dfmF or in the absence of both. Whereas the cells grown in the absence of an unnatural amino acid exhibited no detectable green fluorescence, the cultures containing either tfmF or dfmF were distinctly green. Intact protein mass spectrometry (Figures S10 and S11) and analysis of trypsin digests of the purified sfGFP mutants by MALDI‐MS revealed fragments corresponding to the inclusion of the desired amino acid at the correct position (Figures S13 and S14).

**FIGURE 1:**
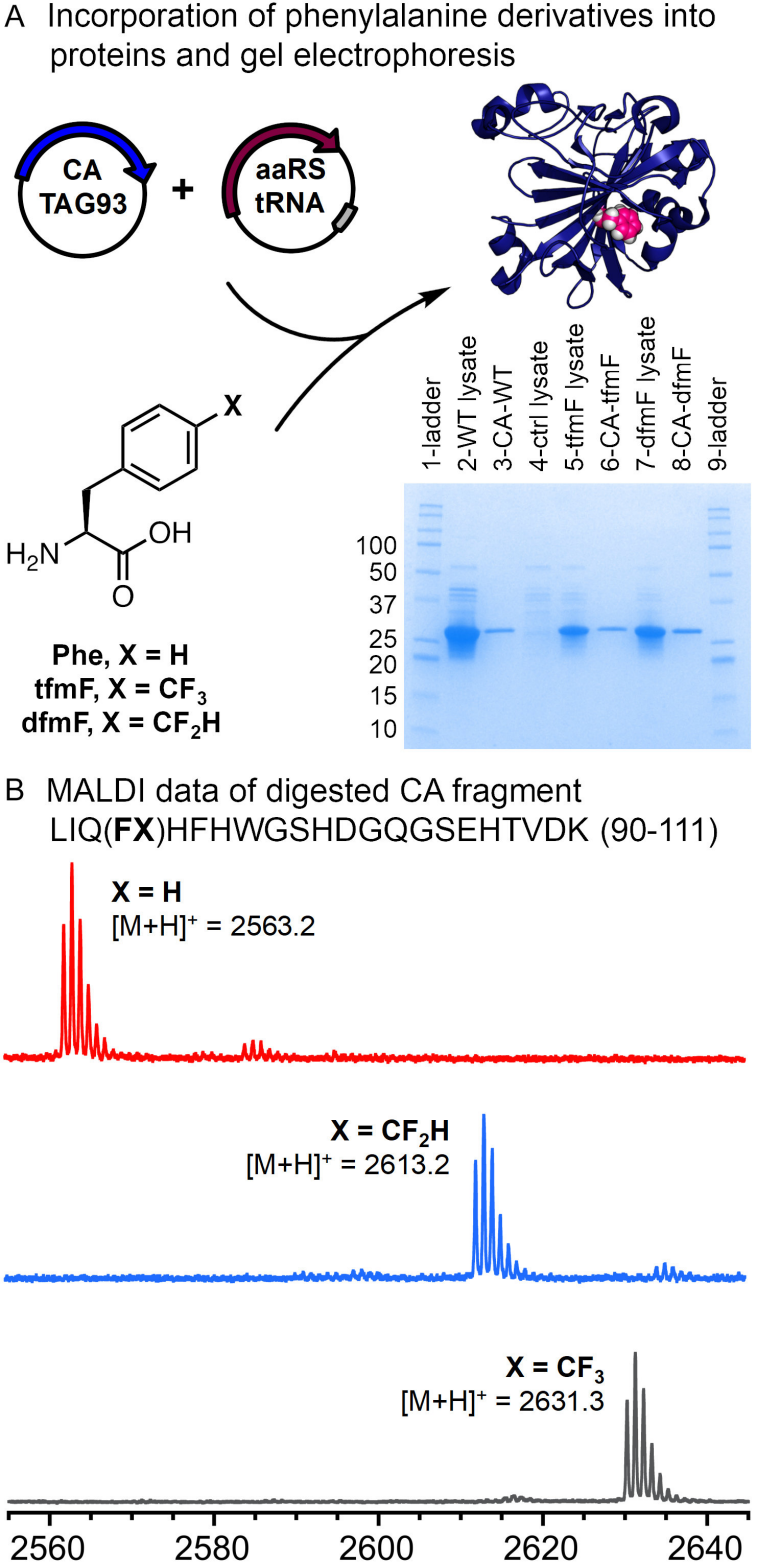
Incorporation of phenylalanine derivatives into proteins using an orthogonal aminoacyl‐tRNA synthetase (aaRS). (A) Approach for incorporating phenylalanine derivatives at position 93 of human carbonic anhydrase II and corresponding gel electrophoresis. (B) MALDI mass spectrometry analysis of digested CA variants, verifying successful incorporation.

We next sought to explore another well‐characterized protein. Here, a thermostable variant of human carbonic anhydrase II (CA) was selected and also engineered with a C‐terminal polyhistidine tag for purification. In addition to the wild‐type protein, CA mutants with amber stop codons at either position 20 or 93 were prepared. These positions are commonly mutated to incorporate noncanonical amino acids. Additionally, substituting phenylalanine at positions 20 and 93 with the structurally related dfmF would likely be conservative mutations. Therefore, the resulting mutants would allow us to survey the behavior of dfmF in solvent‐exposed and buried environments. As with sfGFP, either dfmF or tfmF was successfully incorporated into both proteins (Figures S1 and S2). Representative data from these experiments are shown in Figure 1 for the CA‐93 construct. Gel electrophoresis of the crude lysates indicated overexpression of the desired protein in all cases, except for the control, in which the unnatural amino acids were withheld. In addition to intact protein mass spectrometric studies (Figures S5–S8), digestion of the purified proteins with trypsin and subsequent analysis by MALDI‐MS confirmed the incorporation of the correct amino acid into CA at position 93 (Figures 1B, S16 and S17). Significantly, no mass spectrometry evidence was found in these experiments for misincorporation of either phenylalanine or tyrosine in the CA‐93 mutants.

With these results in hand, the ^19^F NMR spectrum of each fluorinated protein was then examined. Compared to trifluoromethyl‐based probes, which are commonly used in protein ^19^F NMR studies [65, 66, 67, 68, 69, 70, 71, 72], the difluoromethyl group was expected to provide additional spectral information that might be useful for studying biopolymers. Due to the relatively low symmetry of the CF_2_H group, the orientation of the functional group can vary with the local environment, potentially rendering the ^19^F NMR feature a sensitive reporter of the protein structure [45, 73]. Interactions of the highly polarized F_2_C─H bond with other functional groups, including hydrogen bond acceptors, can further perturb the ^19^F NMR signals, possibly providing additional structural insights.

**FIGURE 2:**
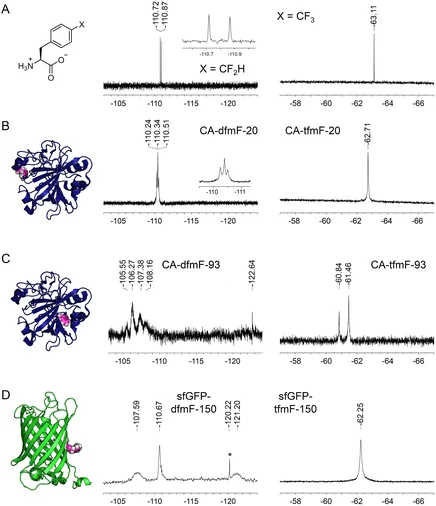
^19^F NMR spectral characterization of 4‐difluoromethyl‐L‐phenylalanine, 4‐trifluoromethyl‐L‐phenylalanine, and proteins containing these amino acids. (A) ^19^F NMR spectra of 20 µM 4‐difluoromethyl‐L‐phenylalanine (dfmF, ^2^ *J*_HF_ = 56.2 Hz) and 100 µM 4‐trifluoromethyl‐L‐phenylalanine (tfmF). (B) ^19^F NMR spectra of 16 µM CA‐dfmF‐20 and 30 µM CA‐tfmF‐20. (C) ^19^F NMR spectra of 8 µM CA‐dfmF‐93 and 20 µM CA‐tfmF‐93. (D) ^19^F NMR spectra of 18 µM sfGFP‐dfmF‐150 and 170 µM sfGFP‐tfmF‐150. All ^19^F NMR spectra of proteins were acquired in 100 mM pH 7.0 sodium phosphate buffer at 298 K, using trifluoroacetic acid in deuterium oxide as an external standard (−76.55 ppm). The ^19^F NMR spectra of dfmF and tfmF were acquired similarly in 1 × pH 7.4 phosphate‐buffered saline. The chemical shifts are shown in ppm along the *X*‐axis. All spectra were recorded without ^1^H decoupling. * indicates the trace amount of the fluoride ion leached from the NMR tube.

**FIGURE 3:**
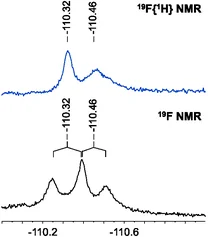
^19^F{^1^H} NMR and ^19^F NMR spectra of CA ‐dfmF‐20.

Our initial investigation was focused on dfmF‐ and tfmF‐labeled CA derivatives. For the CA variant containing dfmF at position 20, three peaks were observed in the ^19^F NMR spectrum (Figure 2B). In the proton‐decoupled ^19^F{^1^H} NMR spectrum, a broad signal (−110.46 ppm) and a relatively sharp signal (−110.32 ppm) were found, suggesting that the observed splitting pattern can be explained as two partially overlapped doublets (Figure 3). Alternatively, these signals could correspond to the two inner components of an AB system with a high ratio of the ^19^F–^19^F coupling constant (*J*) to relative chemical shift (*ν*_0_δ), with the outer lines below the detection limit due to low intensities. These observations indicate that the CF_2_H group or the dfmF side chain resides in two distinct environments. This interpretation is consistent with the reported protein crystal structure, which shows that position 20 is located in a natively unstructured region of the protein. In contrast, the tfmF‐labeled analogs showed a featureless, sharp single peak, providing limited structural information. Further studies focused on CA‐dfmF‐93, in which the dfmF side chain is positioned on a β‐sheet and oriented toward the protein interior. The ^19^F NMR spectrum exhibits four broad signals without discernible ^1^H–^19^F spin–spin couplings (Figure 2C). The separations between the outer and the central signals are 271 and 293 Hz, respectively. Given that these values are close to the typical ^2^ *J*_FF_ coupling constant of approximately 280 Hz, the spectral pattern may be tentatively assigned to two nonequivalent fluorine spins. In contrast, two distinguishable signals were observed with the CA‐tfmF‐93 protein. These more complicated spectral features associated with the CA‐93 variants indicate that the side chain buried in the protein interior may experience more restricted motion, leading to signal decoalescence and line broadening. Similar to observations with the CA‐20 derivative, the CF_2_H group in CA‐dfmF‐93 provides richer spectral information, presumably due to its low symmetry and interactions with nearby functional groups.

In parallel, the ^19^F NMR spectra of sfGFP‐dfmF‐150 and sfGFP‐tfmF‐150 were investigated (Figure 2D). For the CF_2_H group‐labeled GFP, three broad signals were observed. This result indicates that the CF_2_H‐containing side chain either undergoes hindered rotation or slowly exchanges between different conformations on the NMR time scale. The multiple conformers observed can be tentatively attributed to potential interactions between the CF_2_H group and proximal side chains, including Tyr152, Tyr201, and Ser203. In contrast, the tfmF‐containing GFP shows only one relatively broad ^19^F NMR signal, suggesting that the CF_3_ group and the side chain are unlikely to interact strongly with the surrounding residues. Overall, these ^19^F NMR experiments highlight the potential advantages of CF_2_H‐containing amino acids as versatile tools for studying protein structures over conventional CF_3_ group‐based probes.

## 3 Conclusion

We have synthesized a new fluorinated noncanonical amino acid, 4‐difluoromethyl‐L‐phenylalanine, and incorporated it into model proteins, including green fluorescent protein and carbonic anhydrase, via standard genetic code expansion techniques. A permissive tRNA/synthetase pair previously developed for 4‐trifluoromethyl‐L‐phenylalanine was found to allow for efficient incorporation of 4‐difluoromethyl‐L‐phenylalanine. Owing to the low symmetry of the CF_2_H group and its capacity to interact with local environments, the ^19^F NMR signals of this moiety are highly sensitive to protein structural variations. As a result, substantially richer NMR spectroscopic information can be obtained from 4‐difluoromethyl‐L‐phenylalanine‐labeled proteins, underscoring the significant advantages of this new amino acid over its classic trifluoromethyl analog.

## Funding

This study was supported by the National Institute of General Medical Sciences (P20GM103430 and R24GM141526), National Science Foundation (CHE‐2117141), National Center for Research Resources (S10RR025062), and American Chemical Society Petroleum Research Fund (70501‐ND4).

## Conflicts of Interest

The authors declare no conflicts of interest.

## Supporting information

Additional figures and schemes, materials, experimental procedures; characterization data (1D and 2D NMR, LCMS, HRMS) for all small‐molecule compounds; MALDI mass spectrometry of digested proteins; and gel electrophoresis of proteins. The authors have cited additional references within the Supporting Information [74].

## Data Availability

The data that support the findings of this study are available in the Supporting Information of this article.
