# Supplementary material for: Synthesis and In Vivo Incorporation of 4‐Difluoromethyl‐L‐Phenylalanine Into Proteins
Source: Chembiochem. 2026 Sep 23;27(18):e70541. doi: 10.1002/cbic.70541 (PMC13599698; doi:10.1002/cbic.70541)
Supplement: Supplementary file 1 [file CBIC-27-e70541-s001.pdf]

Supporting Information

Synthesis and In Vivo Incorporation of 4-Difluoromethyl-L-Phenylalanine Into Proteins

Daniel S. Honeycutt,[a] Jason M. Mrosla,[b] Sarah A. Sexton,[b] Giovanni E. Cavalli,[b] Emmerson G. Bartels,[b] Brian Pallares,[b] William K. McCarthy,[b] Ruojun Wu,[b] Fang Wang,*[a] Jacob M. Goldberg*[b]

[a] Department of Chemistry, University of Rhode Island, 140 Flagg Rd, Kingston, RI 02881, USA.

[b] Department of Chemistry, Colgate University, 13 Oak Drive, Hamilton, NY 13346, USA.

*To whom correspondence should be addressed
*E-mail: fangwang@uri.edu
*E-mail: jgoldberg@colgate.edu

Table of Contents

General: Chemical Synthesis and Analysis. 2
Chemical Synthesis. 3
Plasmid Information. 5
Bacterial Transformation. 6
Protein Expression. 7
Protein Purification. 8
Gel Electrophoresis. 9
Protein NMR Experiments. 10
Protein Intact Mass Spectrometric Analysis. 11
MALDI. 16
NMR Spectra. 24
References. 41

General: Chemical Synthesis and Analysis.

Reagents were purchased from commercial sources and used as received. Solvents were saturated with argon, purified by passage through two columns of activated alumina, and stored over 3 Å molecular sieves inside an MBraun dry box. Reaction mixtures were monitored by thin-layer chromatography (TLC) on pre-coated, aluminum-backed silica gel 60 F254 plates. Column chromatography was performed on silica gel 60 (230-400 mesh). Unless otherwise specified, all NMR spectra were recorded on a Bruker Avance NMR spectrometer. For CA-dfmF-93, the ^{19}F NMR spectrum was acquired on a Bruker Avance III 600 MHz NMR spectrometer equipped with a TCI cryoprobe. ^{1}H and ^{13}C chemical shifts are reported in ppm relative to $SiMe_4$ (δ = 0.00 ppm). ^{1}H and ^{13}C NMR spectra were referenced internally to residual solvent peaks. ^{19}F spectra were referenced externally to $CFCl_3$ (0.0 ppm). Low-resolution electrospray mass spectra were acquired on a Shimadzu LCMS-2020 spectrometer or an Agilent InfinityLab LC/MSD iQ system. High-resolution mass spectra and MS/MS analyses were acquired on an AB SCIEX TripleTOF 4600 mass spectrometer equipped with a DuoSpray™ ion source. MALDI-MS data were collected with a Bruker autoflex® maX TOF instrument. Milli-Q purified water with resistivity of at least 18 MΩ·cm^{-1} was used to prepare all buffers.

Chemical Synthesis.

***N*-Fmoc-3-iodo-L-alanine methyl ester**

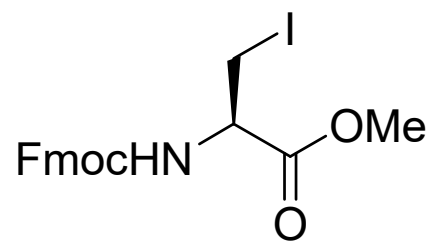

Imidazole (409 mg, 6.00 mmol), Fmoc-L-Ser-OMe (2.05 g, 6.00 mmol), and PPh_3 (1.57 g, 6.00 mmol) were added to a 50 mL Schlenk flask. The flask was purged with N_2 before the addition of anhydrous CH_2Cl_2 (15 mL). After all solids were dissolved, the mixture was chilled to 0 °C using an ice bath. Iodine (1.52 g, 6.00 mmol) was added in three portions under positive N_2 flow with stirring. The mixture was warmed to rt and stirred at the same temperature for 2 h. The precipitate was removed by suction filtration and washed with Et_2O. The filtrate was collected and concentrated under vacuum. The crude product was purified by flash chromatography (silica gel, ethyl acetate:hexanes = 1:2). The resulting solid, which contained a small amount of Fmoc-dehydroalanine methyl ester, was further purified by recrystallization in hot ethyl acetate:hexanes = 1:2 to afford a white solid (1.85 g, 68% yield). ^{1}H NMR (400 MHz, $CDCl_3$) δ 7.78 (d, *J* = 7.5 Hz, 2H), 7.62 (d, *J* = 7.5 Hz, 2H), 7.41 (t, *J* = 7.3 Hz, 2H), 7.33 (tdd, *J* = 7.4, 2.3, 1.1 Hz, 2H), 5.67 (d, *J* = 7.3 Hz, 1H), 4.59 (dt, *J* = 7.4, 3.7 Hz, 1H), 4.42 (ddd, *J* = 23.3, 10.6, 7.3 Hz, 2H), 4.26 (t, *J* = 7.1 Hz, 1H), 3.83 (s, 3H), 3.61 (d, *J* = 3.7 Hz, 2H). MS (ESI) m/z: $[M+H]^+$ Calcd. 452.0, Found 452.0. Spectroscopic data were consistent with reported values.[1]

***N*-Fmoc-4-difluoromethyl-L-phenylalanine methyl ester**

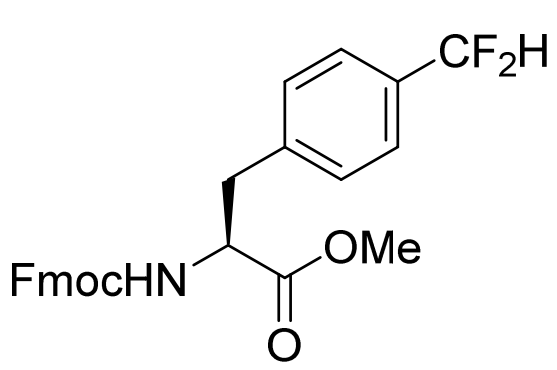

In a glovebox, anhydrous DMF (0.5 mL) was transferred to a 10 mL Schlenk flask containing activated zinc (295 mg, 4.50 mmol) and iodine (56 mg, 0.225 mmol). The resulting yellow mixture was stirred at rt and turned gray in a few minutes. A portion of *N*-Fmoc-3-iodo-L-alanine methyl ester (678 mg, 1.50 mmol) was added to the reaction, followed by anhydrous DMF (2.5 mL) and I_2 (56 mg, 0.225 mmol). After 3 h, $Pd_2(dba)_3$ (71 mg, 0.075 mmol) and SPhos (61 mg, 0.150 mmol) were added. The flask was transferred out of the glovebox and attached to a Schlenk line under N_2. An aliquot of *p*-bromo-α,α-difluorotoluene (258 µL, 2.00 mmol) was added to the flask via a syringe. The reaction mixture was stirred at 50 °C overnight. The reaction mixture was cooled to rt. The insoluble material was removed by filtration through Celite® and rinsed with DMF (20 mL). The filtrate was diluted with water (150 mL) and extracted with ethyl acetate (50 mL × 3). The combined organic layer was washed with water (100 mL × 2) and dried over Na_2SO_4. The solvents were removed under vacuum. The crude product was purified by column chromatography (silica gel, ethyl acetate:hexanes = 1:3) to afford a tan solid (454 mg, 67% yield). ^{1}H NMR (400 MHz, $CDCl_3$) δ 7.78 (d, *J* = 7.6 Hz, 2H), 7.57 (dd, *J* = 7.5, 4.5 Hz, 2H), 7.41 – 7.39 (m, 4H), 7.32 (t, *J* = 7.5 Hz, 2H), 7.17 (d, *J* = 7.8 Hz, 2H), 6.62 (t, *J* = 55.8 Hz, 1H), 5.25 (d, *J* = 8.1 Hz, 1H), 4.69 (q, *J* = 6.2 Hz, 1H), 4.47 (dd, *J* = 10.7, 7.1 Hz, 1H), 4.38 (dd, *J* = 10.7, 6.8 Hz, 1H), 4.21 (t, *J* = 7.0 Hz, 1H), 3.74 (s, 3H), 3.20 (dd, *J* = 13.9, 5.7 Hz, 1H), 3.13 (dd, *J* = 13.8, 5.9 Hz, 1H). ^{19}F NMR (376 MHz, $CDCl_3$) δ −111.60 (d, *J* = 56.5 Hz), ^{13}C NMR (101 MHz, $CDCl_3$) δ 171.75, 155.62, 143.92, 143.80, 141.50, 138.79, 133.40 (t, *J* = 22.4 Hz), 129.80, 127.90, 127.21, 125.96 (t, *J* = 5.9 Hz), 125.20, 125.12, 120.18, 114.70 (d, *J* =

238.8 Hz), 67.05, 54.74, 52.64, 47.30, 38.15. MS (ESI) m/z: $[M+Na]^+$ Calcd. 474.1, Found 474.1. HRMS (ESI) m/z: $[M+H]^+$ Calcd. for $C_{26}H_{24}F_2NO_4^+$; 452.1668, Found 452.1646.

N-Fmoc-4-difluoromethyl-L-phenylalanine

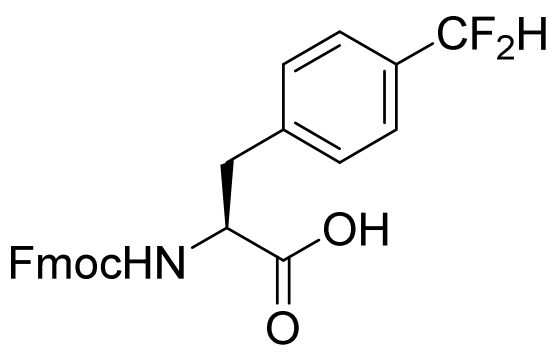

In a round-bottom flask, *N*-Fmoc-4-difluoromethyl-L-phenylalanine methyl ester (226 mg, 0.50 mmol) was dissolved in THF (1.0 mL). The reaction was cooled to 0 °C in an ice bath before the addition of 1 M LiOH aq. (1.0 mL, 1.0 mmol). The mixture was stirred at 0 °C. The progress of the reaction was monitored by TLC. Upon completion of the reaction (1 h), an aliquot of 2 M HCl aq. (0.6 mL) was added. The mixture was extracted with ethyl acetate (15 mL × 4). The combined organic layer was dried over Na_2SO_4 and concentrated under vacuum. The crude product was purified by column chromatography (silica gel, ethyl acetate:hexanes:HOAc = 20:20:1) to afford a white solid (174 mg, 80%). ^1H NMR (400 MHz, CD_3OD) δ 7.79 (d, J = 7.6 Hz, 2H), 7.59 (d, J = 6.1 Hz, 2H), 7.44 (d, J = 7.9 Hz, 2H), 7.38 (t, J = 7.7 Hz, 2H), 7.35 (d, J = 7.9 Hz, 2H), 7.29 (t, J = 7.4 Hz, 2H), 6.70 (t, J = 56.5 Hz, 1H), 4.44 (dd, J = 9.7, 4.7 Hz, 1H), 4.28 (dd, J = 23.2, 10.6 Hz, 1H), 4.27 (dd, J = 22.7, 10.3 Hz, 1H), 4.15 (t, J = 6.9 Hz, 1H), 3.27 (dd, J = 13.7, 4.6 Hz, 1H), 3.00 (dd, J = 13.9, 9.6 Hz, 1H). ^{13}C NMR (101 MHz, CD_3OD) δ 174.90, 158.38, 145.23, 145.19, 142.56, 141.79, 134.57 (t, J = 21.9 Hz) 130.72, 128.76, 128.14, 126.71 (t, J = 6.0 Hz), 126.27, 126.20, 120.89, 116.35 (t, J = 236.6 Hz), 67.92, 56.57, 48.32, 38.30. ^{19}F NMR (376 MHz, CD_3OD) δ −111.50 (d, J = 53.2 Hz). MS (ESI) m/z: $[M+H]^+$ Calcd. 438.2, Found 438.2. HRMS (ESI) m/z: $[M+H]^+$ Calcd. for $C_{25}H_{22}F_2NO_4^+$; 438.1512, Found 438.1506.

4-Difluoromethyl-L-phenylalanine

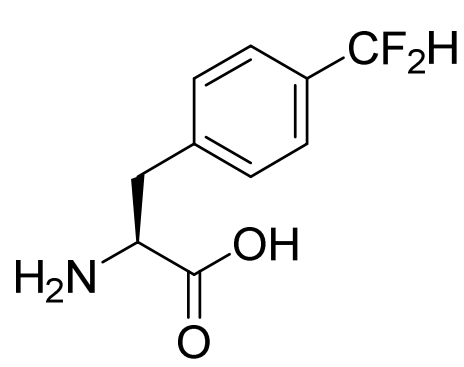

In a round-bottom flask, *N*-Fmoc-4-difluoromethyl-L-phenylalanine (219 mg, 0.50 mmol) was suspended in acetonitrile (4 mL). An aliquot of 4-methylpiperidine (400 μL) was added. The reaction mixture was stirred for 5 min and allowed to stand without stirring for 1 h. The solvent was removed under vacuum. The resulting solid was suspended in CH_2Cl_2 (10 mL) and isolated by suction filtration. The solid was washed with additional CH_2Cl_2 (20 mL) and dried under vacuum. A white solid was obtained (78 mg, 72%). ^1H NMR (400 MHz, CD_3OD) δ 7.53 (d, J = 8.1 Hz, 2H), 7.44 (d, J = 8.1 Hz, 2H), 6.75 (t, J = 56.3 Hz, 1H), 3.82 (dd, J = 8.6, 4.6 Hz, 1H), 3.36 (dd, J = 14.6, 4.5 Hz, 1H), 3.08 (dd, J = 14.5, 8.6 Hz, 1H). ^{19}F NMR (376 MHz, CD_3OD) δ −111.80 (d, J = 56.3 Hz). ^{13}C NMR (101 MHz, CD_3OD) δ 173.46, 140.31, 135.23 (t, J = 22.4 Hz), 130.88, 127.23 (t, J = 6.0 Hz), 116.28 (t, J = 236.6 Hz), 57.33, 38.01. MS (ESI) m/z: $[M+H]^+$ Calcd. 216.1, Found 215.9. HRMS (ESI) m/z: $[M+H]^+$ Calcd. for $C_{10}H_{12}F_2NO_2^+$; 216.0831, Found 216.0827.

Plasmid Information.

pDule-tfmF A65V S158A was a gift from Ryan Mehl (Addgene plasmid # 85484; http://n2t.net/addgene:85484; RRID:Addgene_85484).[54] pBad-sfGFP was a gift from Ryan Mehl (Addgene plasmid # 85482; http://n2t.net/addgene:85482; RRID:Addgene_85482).[54] pBad-sfGFP 150TAG was a gift from Ryan Mehl (Addgene plasmid # 85483; http://n2t.net/addgene:85483; RRID:Addgene_85483).[54] pBad-CA was a gift from Ryan Mehl (Addgene plasmid # 105665; http://n2t.net/addgene:105665; RRID:Addgene_105665). pBad-CA TAG20 was a gift from Ryan Mehl (Addgene plasmid # 105666; http://n2t.net/addgene:105666; RRID:Addgene_105666). pBad-CA TAG93 was a gift from Ryan Mehl (Addgene plasmid # 105667; http://n2t.net/addgene:105667; RRID:Addgene_105667)

Plasmid DNA was obtained with a Monarch® Plasmid Miniprep Kit (New England Biolabs #T1010). Plasmid DNA concentrations were determined with a Thermo Scientific Nanodrop 2000C spectrophotometer. Working antibiotic concentrations for each plasmid are listed in **Table S1**.

Table S1. Plasmid Antibiotic Resistance.

Plasmid	Antibiotic Resistance (working concentration)
pDule-tfmF-A65V-S158A	tetracycline (25 µg/mL)
pBad-sfGFP	ampicillin (100 µg/mL)
pBad-sfGFP-150TAG	ampicillin (100 µg/mL)
pBad-CA	ampicillin (100 µg/mL)
pBad-CA TAG20	ampicillin (100 µg/mL)
pBad-CA TAG93	ampicillin (100 µg/mL)

Bacterial Transformation.

NEB® 10-beta electrocompetent *E. coli* cells (New England Biolabs #C3020K) were transformed with the appropriate plasmids by electroporation according to the manufacturer's protocol. Briefly, for a typical electroporation experiment, a portion of thawed cells (25 μL) was combined with 0.1 μg of each DNA plasmid in a sterile microcentrifuge tube. The mixture was transferred to a chilled electroporation cuvette with a 1 mm gap. Electroporation was conducted with a BTX Electro Cell Manipulator 600 at 2.5 kV and 186 Ω. Time constants for electroporation ranged from ca. 1-12 ms. Immediately following electroporation, cells were resuspended in 975 μL of pre-warmed NEB® 10-beta/stable outgrowth medium (New England Biolabs #B9035S) at 37 ºC. The resulting suspension was transferred to a sterile 100 × 17 mm round-bottom culture tube and incubated at 37 ºC for 1 h with shaking at 225 rpm. Aliquots (50-200 μL) of the suspension were spread on pre-warmed LB agar selection plates containing the appropriate antibiotics (**Table S1**) and the plates were incubated at 37 ºC overnight. Single colonies were picked to inoculate cultures in LB media (6 mL) containing antibiotics, as needed. Cultures were grown overnight at 37 ºC with shaking at 225 rpm. Glycerol stocks of transformed cells were established and stored at –80 ºC.

Protein Expression.

Proteins were expressed using an arabinose autoinduction protocol.[74] The general procedure for a 100-mL scale expression is listed here; smaller or larger cultures were scaled accordingly. In a 500 mL baffled flask, sterile aqueous solutions of glycerol (10% v/v; 5 mL), glucose (40% w/v; 120 µL), arabinose (20% w/v; 250 µL), 50× salts (1.25 M Na_2HPO_4, 1.25 M KH_2PO_4, 2.5 M NH_4Cl, 0.25 M Na_2SO_4; 2 mL), $MgSO_4$ (1 M; 200 µL), L-aspartate (5% w/v, pH 7.5; 5 mL), L-leucine (4 mg/mL, pH 7.5; 2 mL), 18 amino acid mix (1 g each of L-alanine, L-arginine hydrochloride, L-asparagine, L-aspartic acid, glycine, L-glutamic acid monosodium salt, L-glutamine, L-histidine hydrochloride, L-isoleucine, L-leucine, L-lysine hydrochloride, L-methionine, L-phenylalanine, L-proline, L-serine, L-threonine, L-tryptophan, and L-valine in H_2O to a total volume of 200 mL; 4 mL), and trace metals ($CaCl_2$ (2 M, 500 µL, in water), $MnCl_2$ (1 M, 500 µL, in water), $ZnSO_4$ (1 M, 500 µL, in water), $CoCl_2$ (0.2 M, 500 µL, in water), $CuCl_2$ (0.2 M, 500 µL, in water), $NiCl_2$ (0.2 M, 500 µL, in water), Na_2MoO_4 (0.2 M, 500 µL, in water), Na_2SeO_3 (0.2 M, 500 µL, in water), H_3BO_3 (0.2 M, 500 µL, in water) and $FeCl_3$ (0.1 M, 25 mL, in 0.1 M HCl) and diluting the resulting mixture to a volume of 50 mL with sterile water. 20 µL) were combined and brought to 100 mL with sterile water. The medium was warmed at 37 ºC for 30 min before antibiotics were added, as needed, according to **Table S1** and then inoculated with 500 µL of an overnight starter culture (6 mL, LB + antibiotics) grown from a freshly streaked colony. The cultures were incubated at 37 ºC for 30 min with shaking at 225 rpm, at which time unnatural amino acids (0.100 mmol; tfmF, 23.4 mg or dfmF, 21.6 mg) freshly dissolved in 8 M NaOH aq. (~16 µL) were added, as needed, to achieve a final concentration of 1 mM. The cultures were incubated at 37 ºC for an additional 24 h with shaking at 225 rpm before harvesting. Cells were harvested by centrifugation at 8,000 × *g* for 20 min at 4 ºC.

Protein Purification.

Cell pellets were resuspended in BugBuster® HT Protein Extraction Reagent (Millipore-Sigma 70922, 5 mL/gram cell pellet) and incubated at room temperature for 20 min on a rotisserie. The crude lysate was clarified by centrifugation at 16,100 × *g* for 20 min. The supernatant was decanted and combined with an equal volume of equilibration buffer (300 mM KCl, 50 mM KH_2PO_4, and 5 mM imidazole). The resulting solution was loaded onto a pre-equilibrated Ni-NTA HisBind® Resin (Millipore-Sigma 70666) gravity filtration column. The resin bed was washed 3× with 4 bed volumes of wash buffer (300 mM KCl, 50 mM KH_2PO_4, and 10 mM imidazole). Elution buffer (300 mM KCl, 50 mM KH_2PO_4, and 250 mM imidazole) was added in small portions and collected in fractions. The flow-through, washes, and eluted fractions were assessed by gel electrophoresis. Fractions containing pure protein, as assessed by gel electrophoresis, were combined and concentrated using Corning Spin-X® UF Concentrators (5,000 or 10,000 MWCO). The elution buffer was exchanged three times for a sodium phosphate buffer (100 mM, pH 7.0). Protein concentrations were determined using the extinction coefficient of sfGFP $\varepsilon_{488\ nm} = 8.33 \times 10^4\ M^{-1}cm^{-1}$ or a Pierce™ Coomassie Bradford Protein Assay Kit (Thermo Scientific #23200).[55] Purified proteins were stored at 4 ºC.

The yield of sfGFP-150-dfmF is 26 mg/L.

The yield of sfGFP-150-tfmF is 123 mg/L.

Gel Electrophoresis.

Samples (5 μL) were combined with 2× Laemmli sample buffer (Bio-Rad 1610737; 4.75 μL) and β-mercaptoethanol (0.25 μL) and denatured at 95 ºC for 5 min. The resulting mixtures were analyzed by SDS-PAGE (Bio-Rad, Any kD™ Mini-PROTEAN® TGX Stain-Free™ protein gels, 4568126) according to the manufacturer's directions. Gels were visualized by staining with Coomassie Brilliant Blue R-250 dye.

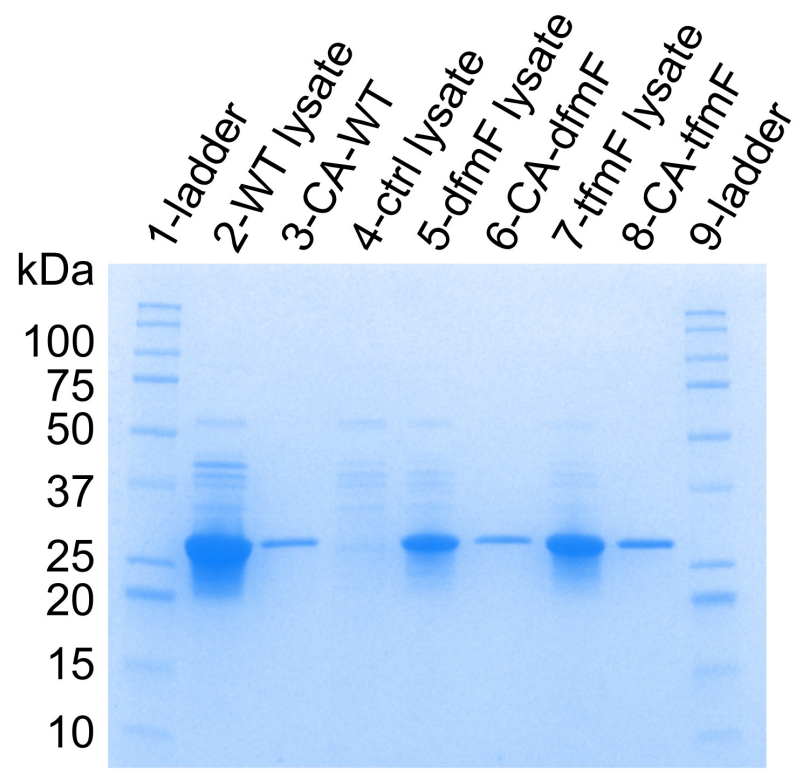

Figure S1. SDS-PAGE analysis of **CA-93**. Lane 2: CA-WT lysate. Lane 3: CA-WT at stock concentration. Lane 4: CA control lysate. Lane 5: CA-dfmF-93 lysate. Lane 6: CA-dfmF-93 at stock concentration. Lane 7: CA-tfmF-93 lysate. Lane 8: CA-dfmF-93 at stock concentration. Ladder: Precision Plus Protein™ Kaleidoscope ™ Prestained Protein Standards (Bio-Rad 1610375).

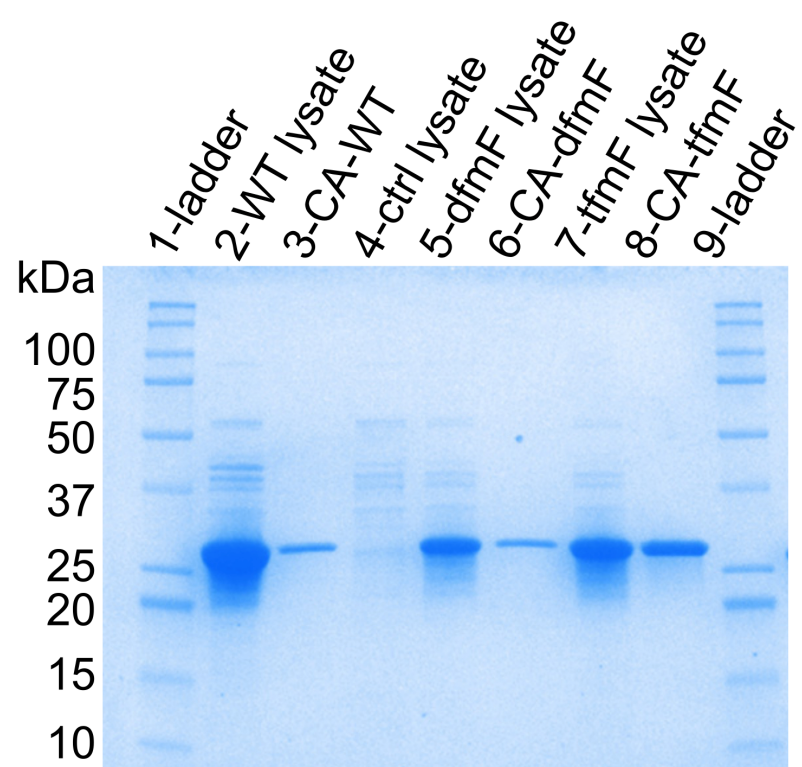

Figure S2. SDS-PAGE analysis of **CA-20**. Lane 2: CA-WT lysate. Lane 3: CA-WT at stock concentration. Lane 4: CA control lysate. Lane 5: CA-dfmF-20 lysate. Lane 6: CA-dfmF-20 at stock concentration. Lane 7: CA-tfmF-20 lysate. Lane 8: CA-dfmF-20 at stock concentration. Ladder: Precision Plus Protein™ Kaleidoscope ™ Prestained Protein Standards (Bio-Rad 1610375).

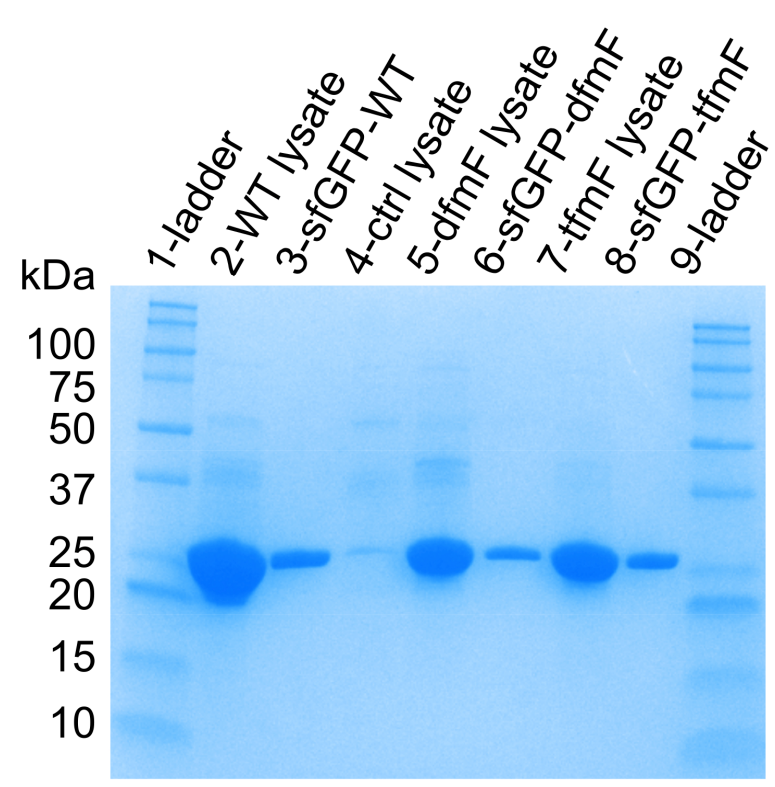

Figure S3. SDS-PAGE analysis of **sfGFP-TAG150**. Lane 2: sfGFP-WT lysate. Lane 3: sfGFP-WT. Lane 4: sfGFP-TAG150 lysate control. Lane 5: sfGFP–TAG150 dfmF lysate. Lane 6: sfGFP–TAG150 dfmF. Lane 7: sfGFP–TAG150 tfmF lysate. Lane 8: sfGFP–TAG150 tfmF. Ladder: Precision Plus Protein™ Kaleidoscope ™ Prestained Protein Standards (Bio-Rad 1610375).

Protein NMR Experiments.

For each protein sample, ^{19}F NMR data were collected using a Bruker Avance 400 MHz NMR spectrometer (376.028 MHz). Standard decoupling parameters were used with a 100000 Hz spectral width, 18 μs pulse length, 6.5 μs pre-scan delay, and 1.00 s relaxation delay.

All spectra were recorded at 25 °C in pH 7.0 sodium phosphate buffer (100 mM) with a coaxial insert for 5 mm NMR (Wilmad, catalog number: WGS-5BL-SP), which contained trifluoroacetic acid (3.3 μM) in D_2O.

Protein Intact Mass Spectrometric Analysis.

CA

MAHHWGYGKH NGPEHWHKDF PIAKGERQSP VDIDTHTAKY DPSLKPLSVS YDQATSLRIL NNGHTFNVEF DDSQDKAVLK GGPLDGTYRL IQFHFHWGSH DGQGSEHTVD KKKYAAELHL VHWNTKYGDF GKAVQQPDGL AVLGIFLKVG SANPGLQKVV DVLDSIKTKG KSADFTNFDP RGLLPESLDY WTYPGSLTTP PLLECVTWIV LKEPISVSSE QVSKFRKLNF NGEGEPEEPM VDNWRPTQPL KNRQIKASFK HHHHHH

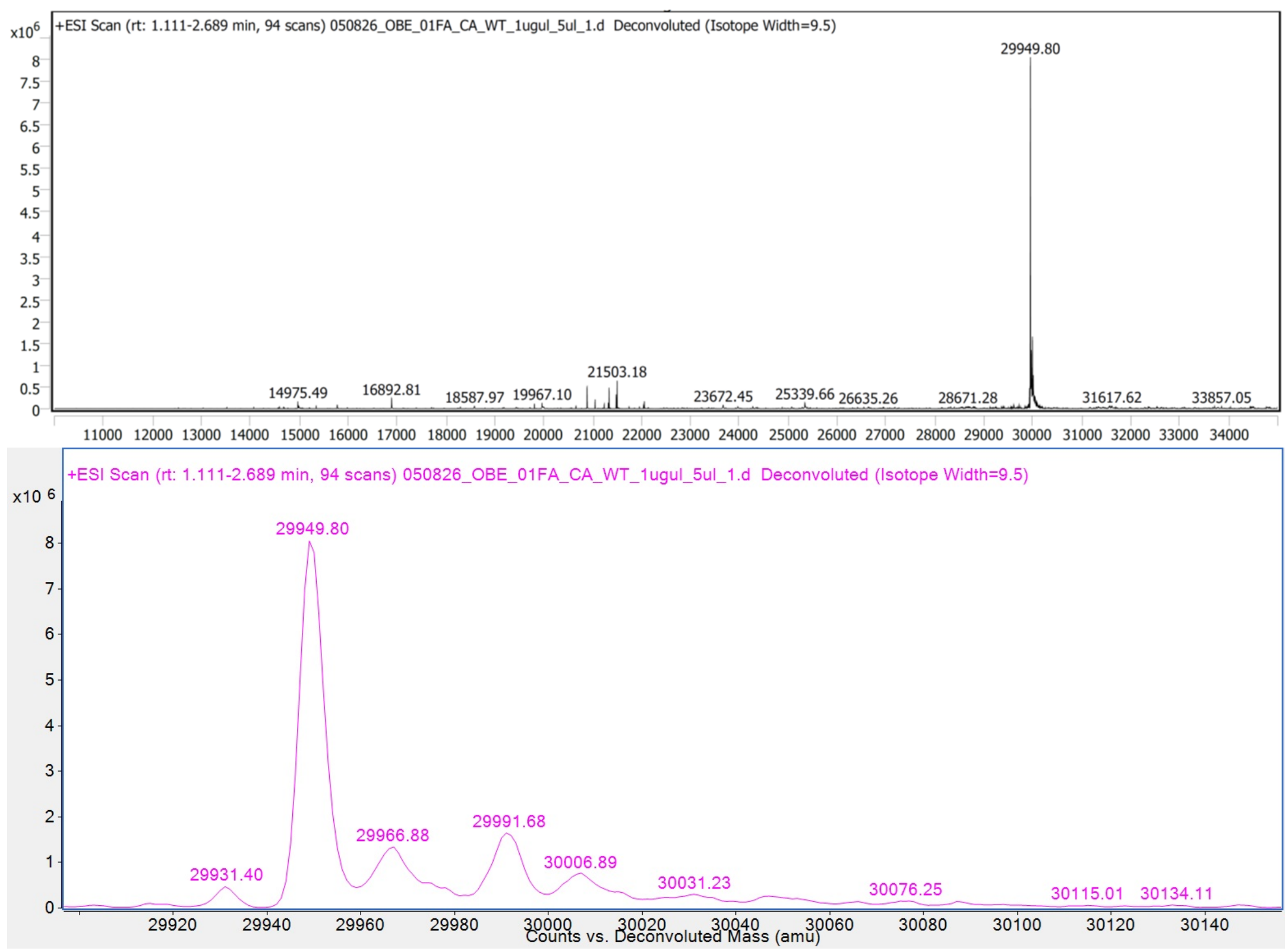

Figure S4. Intact mass spectrometric data of CA-WT. Predicted 30081; Found 29950, corresponding to the loss of one methionine residue.

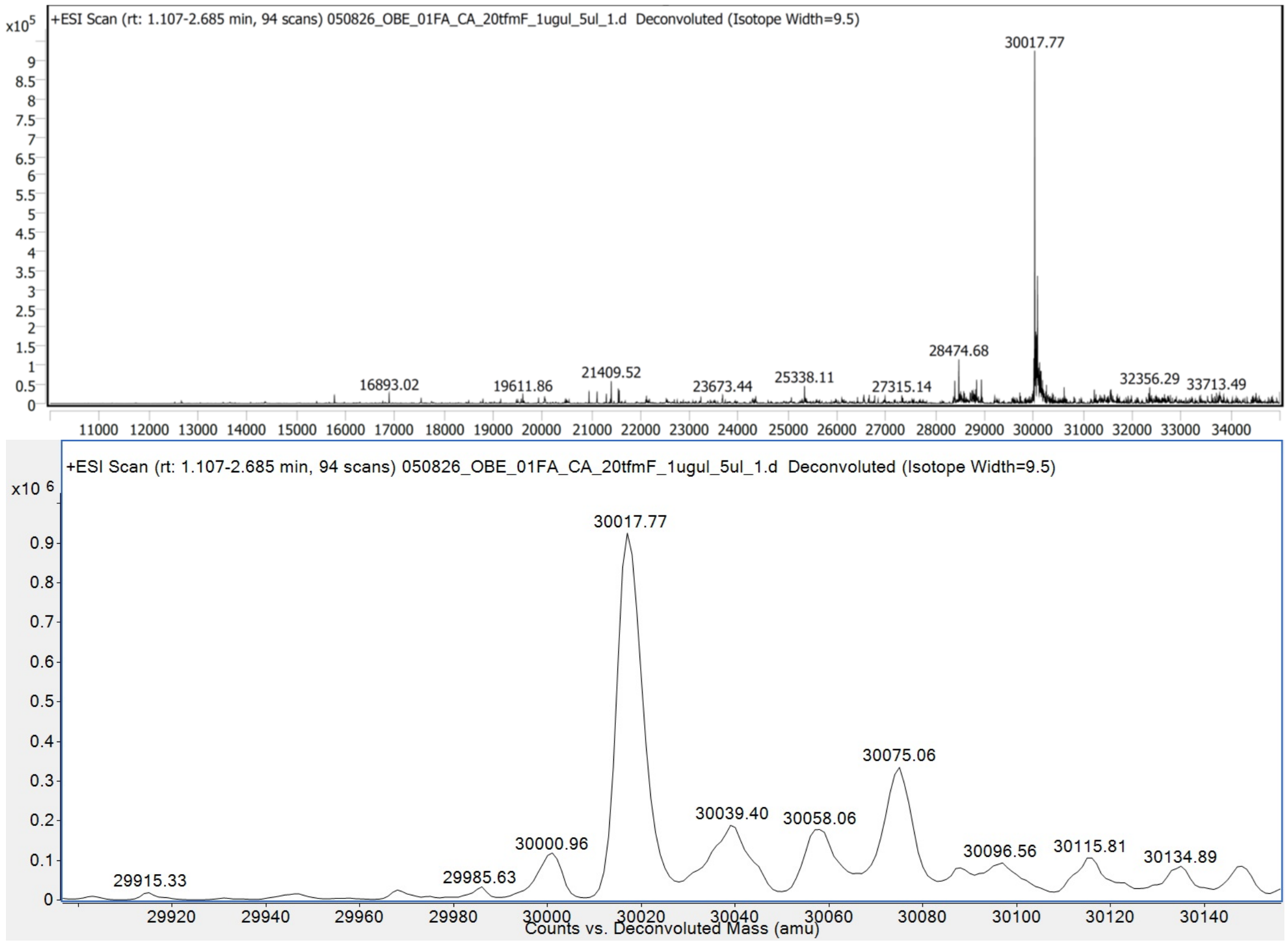

Figure S5. Intact mass spectrometric data of CA-tmfF-20. Predicted 30149; Found 30018, corresponding to the loss of one methionine residue.

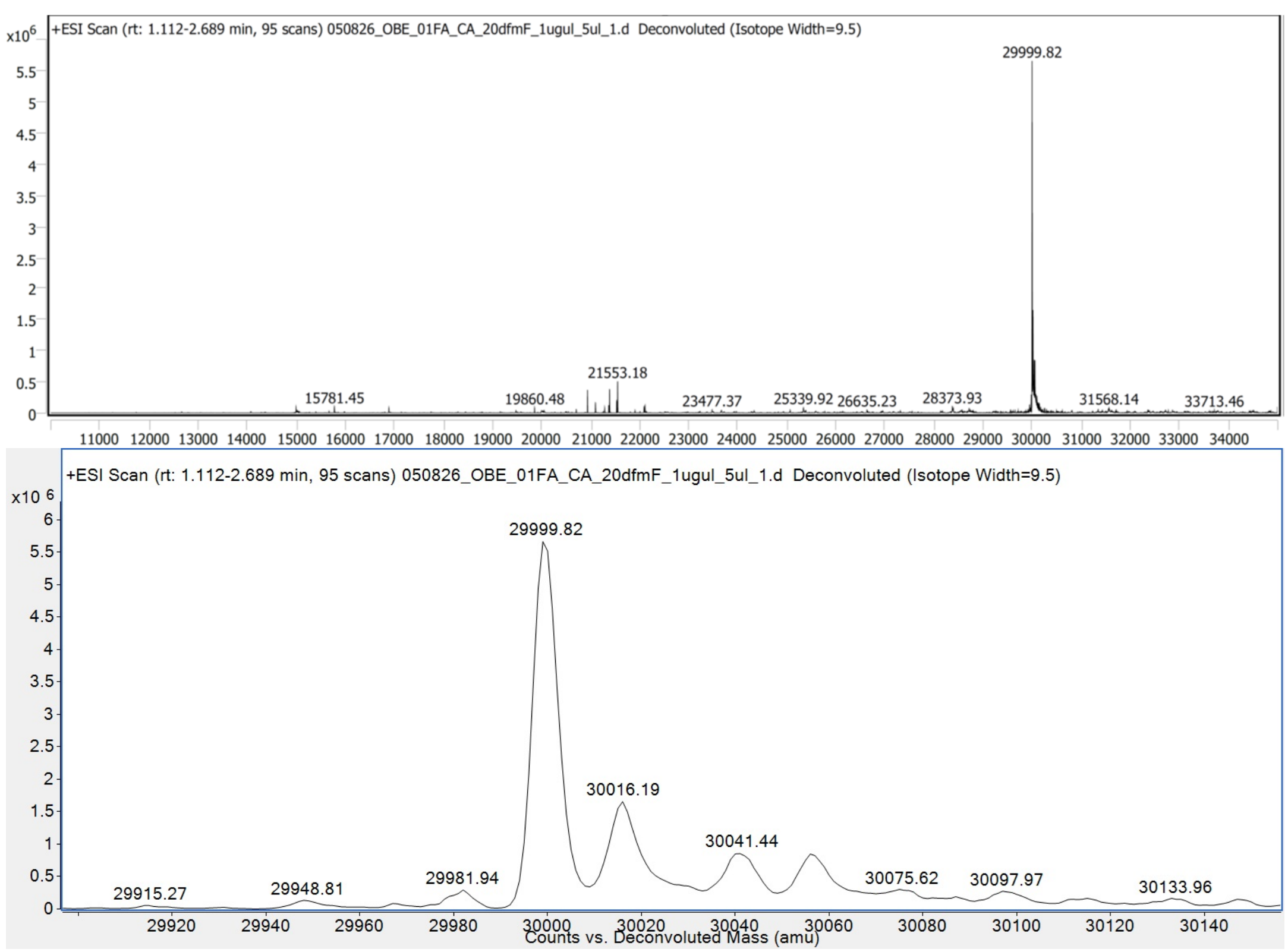

Figure S6. Intact mass spectrometric data of CA-dmfF-20. Predicted 30131; Found 30000, corresponding to the loss of one methionine residue.

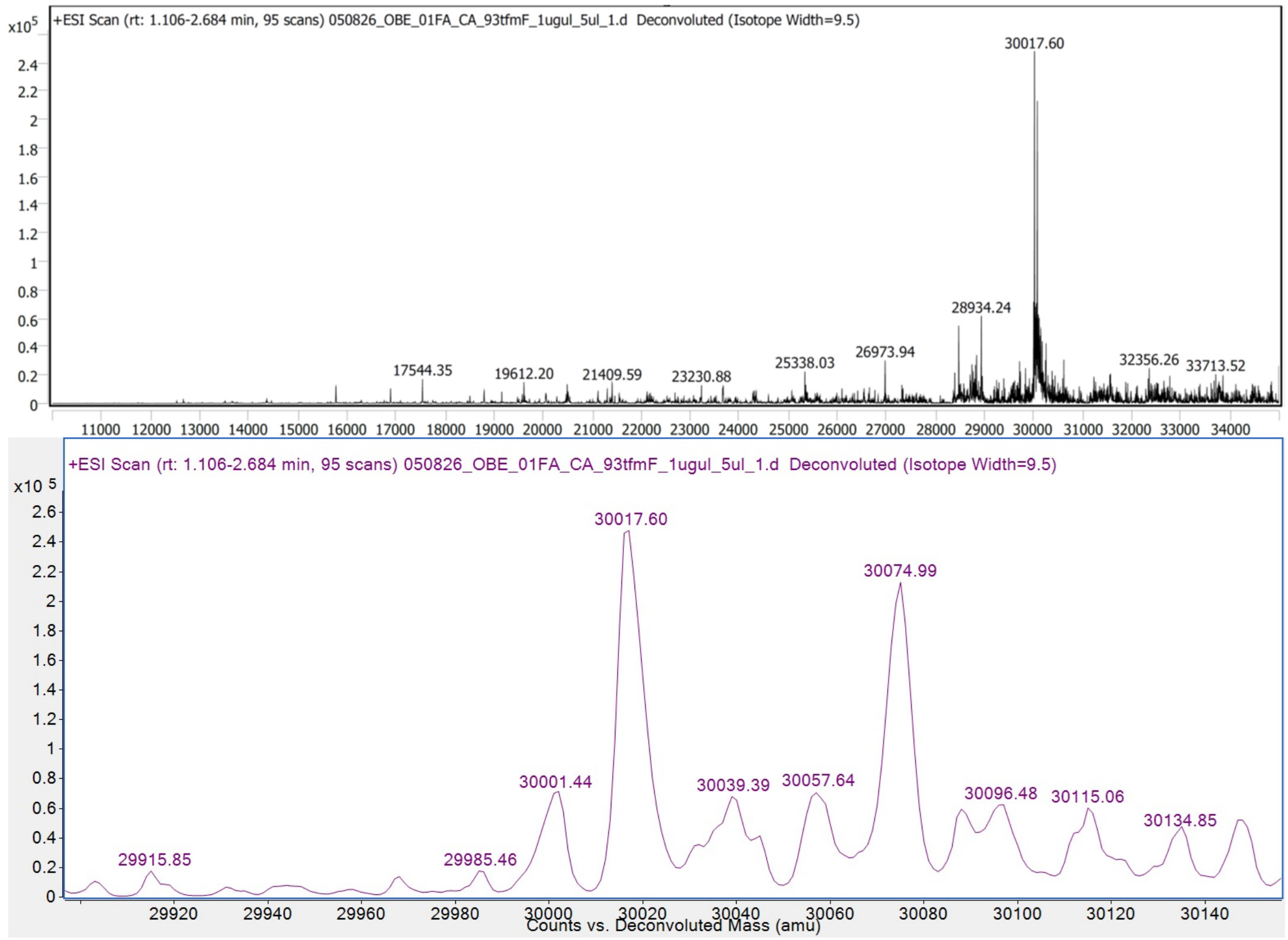

Figure S7. Intact mass spectrometric data of CA-tmfF-93. Predicted 30149; Found 30018, corresponding to the loss of one methionine residue.

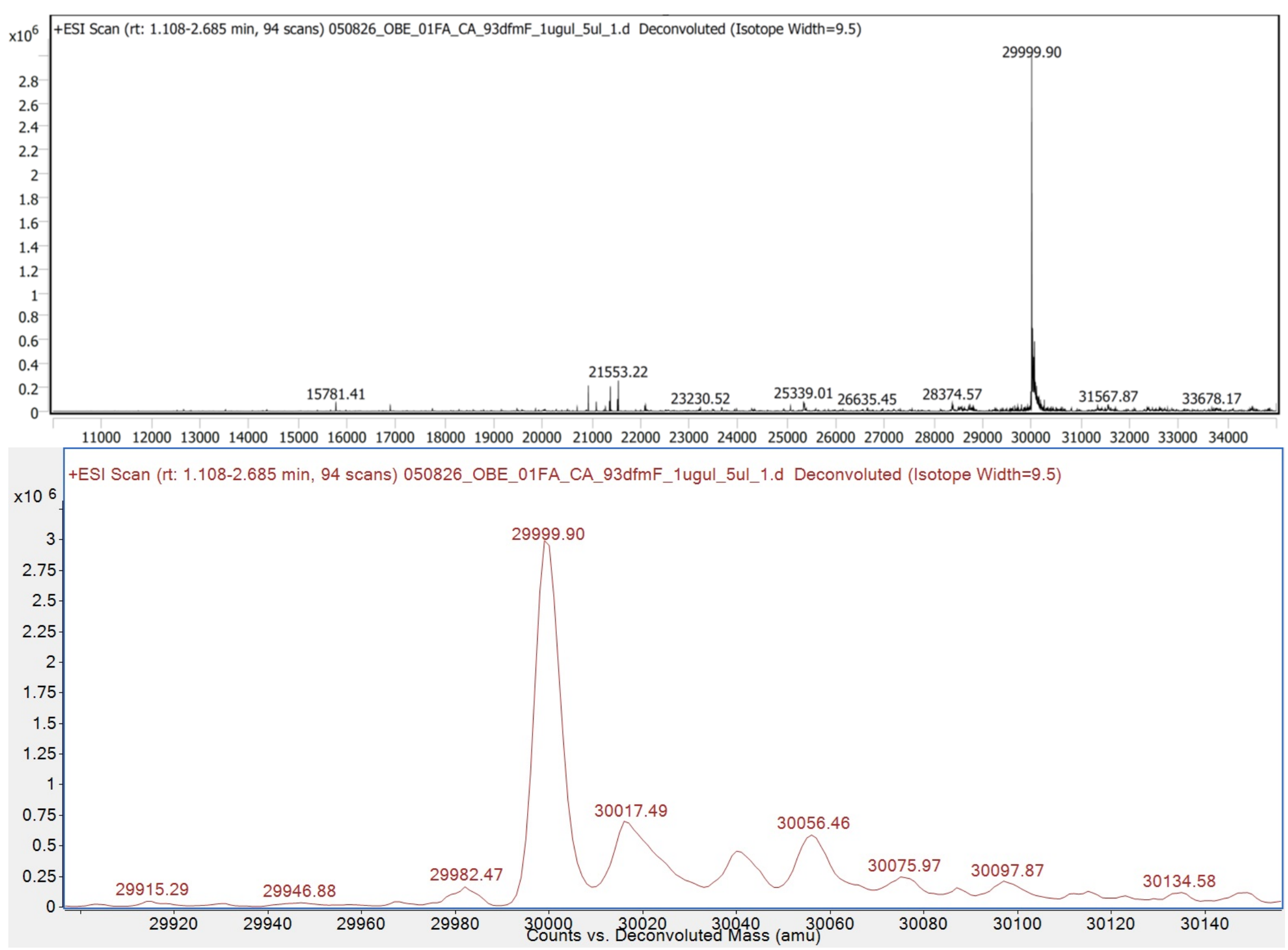

Figure S8. Intact mass spectrometric data of CA-dmfF-93. Predicted 30131; Found 30000, corresponding to the loss of one methionine residue.

sfGFP

MVSKGEELFT GVVPILVELD GDVNGHKFSV RGEGEGDATN GKLTLKFICT TGKLPVPWPT LVTTLTYGVQ CFSRYPDHMK RHDFFKSAMP EGYVQERTIS FKDDGTYKTR AEVKFEGDTL VNRIELKGID FKEDGNILGH KLEYNFNSHN VYITADKQKN GIKANFKIRH NVEDGSVQLA DHYQQNTPIG DGPVLLPDNH YLSTQSVLSK DPNEKRDHMV LLEFVTAAGI THGMDELYKG SHHHHHH

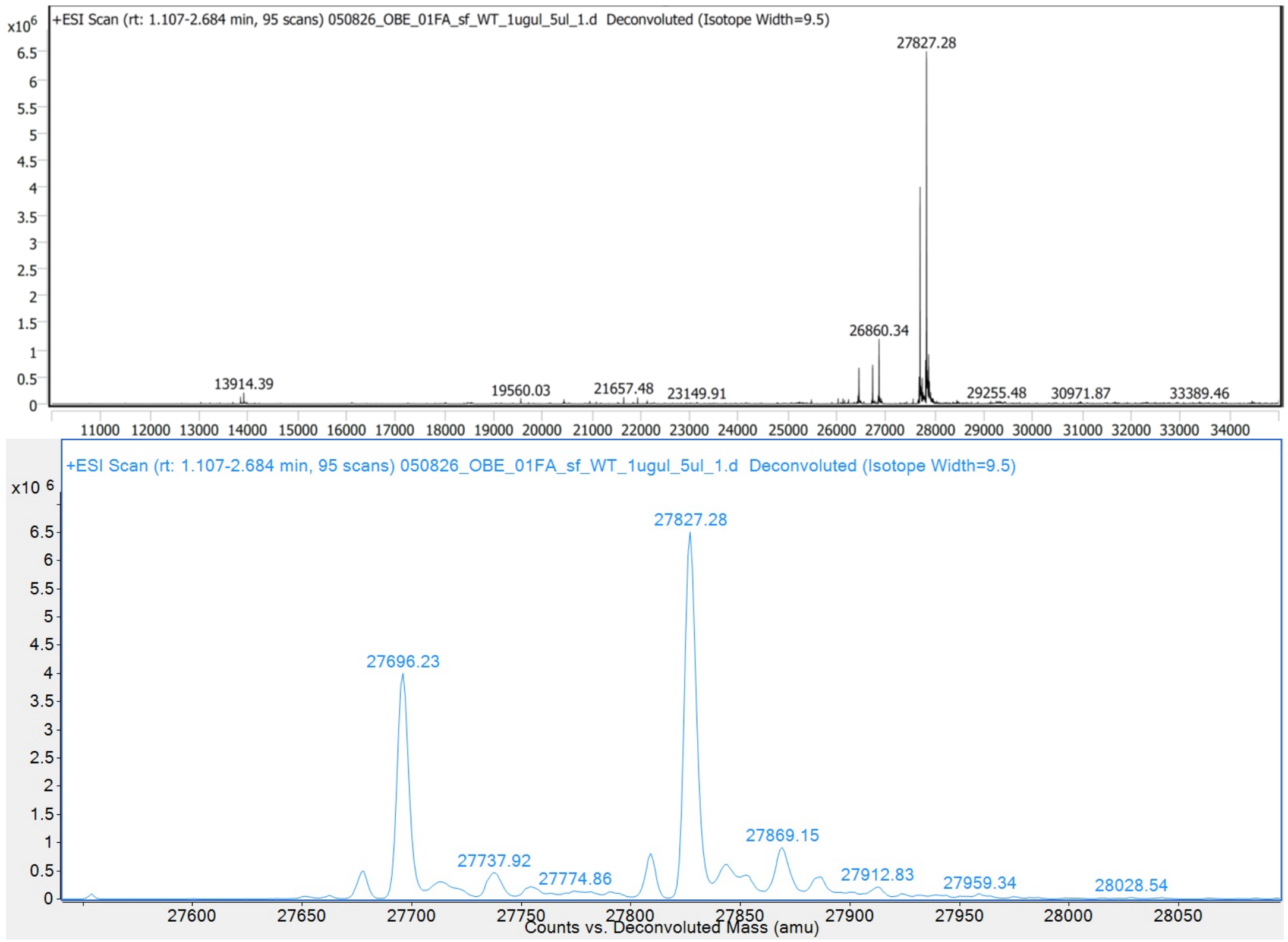

Figure S9. Intact mass spectrometric data of sfGFP. Predicted 27827; Found 27827, and 27696 corresponding to the loss of one methionine residue.

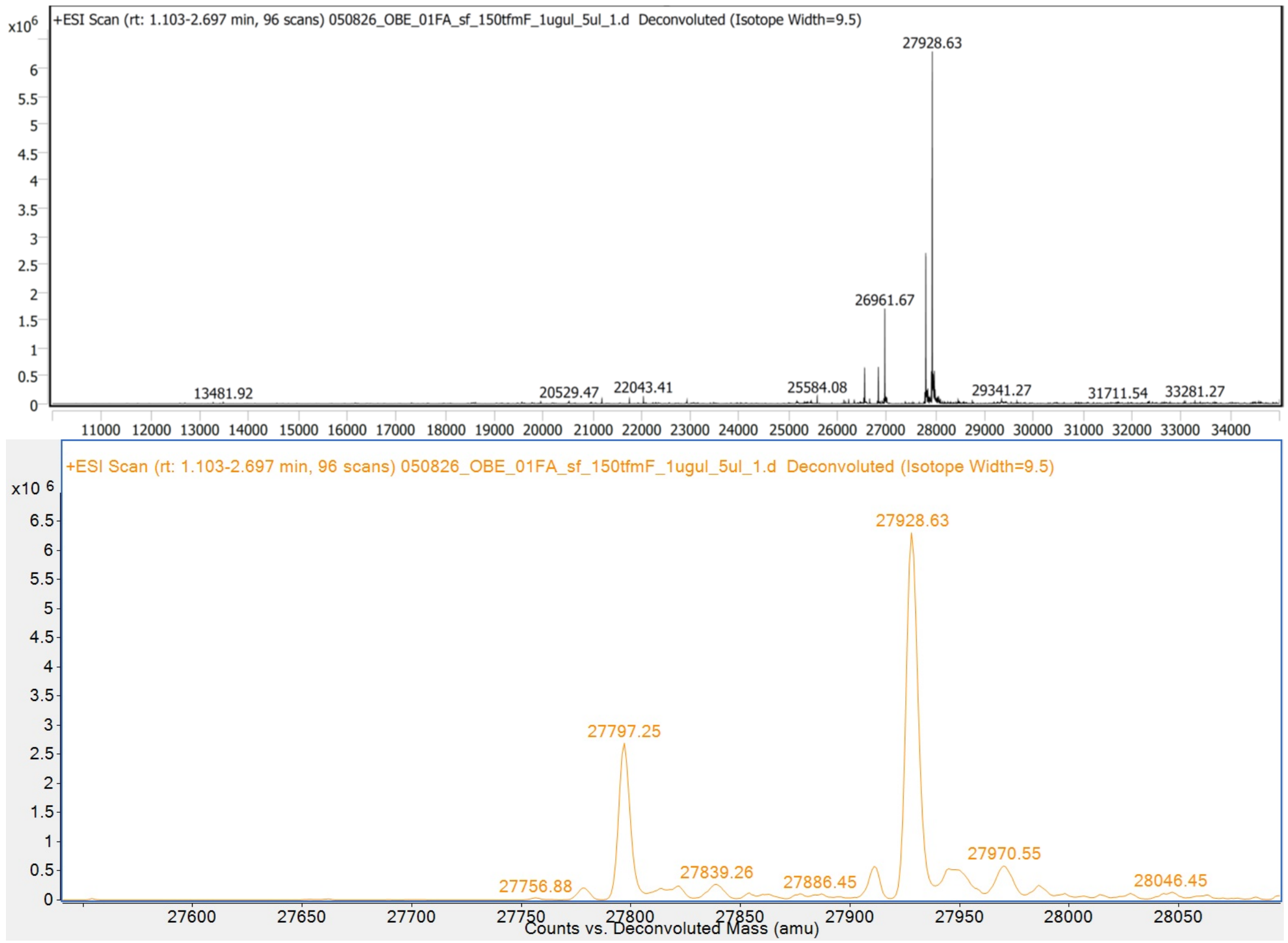

Figure S10. Intact mass spectrometric data of sfGFP-tfmF-150. Predicted 27928; Found 27929, and 27797 corresponding to the loss of one methionine residue.

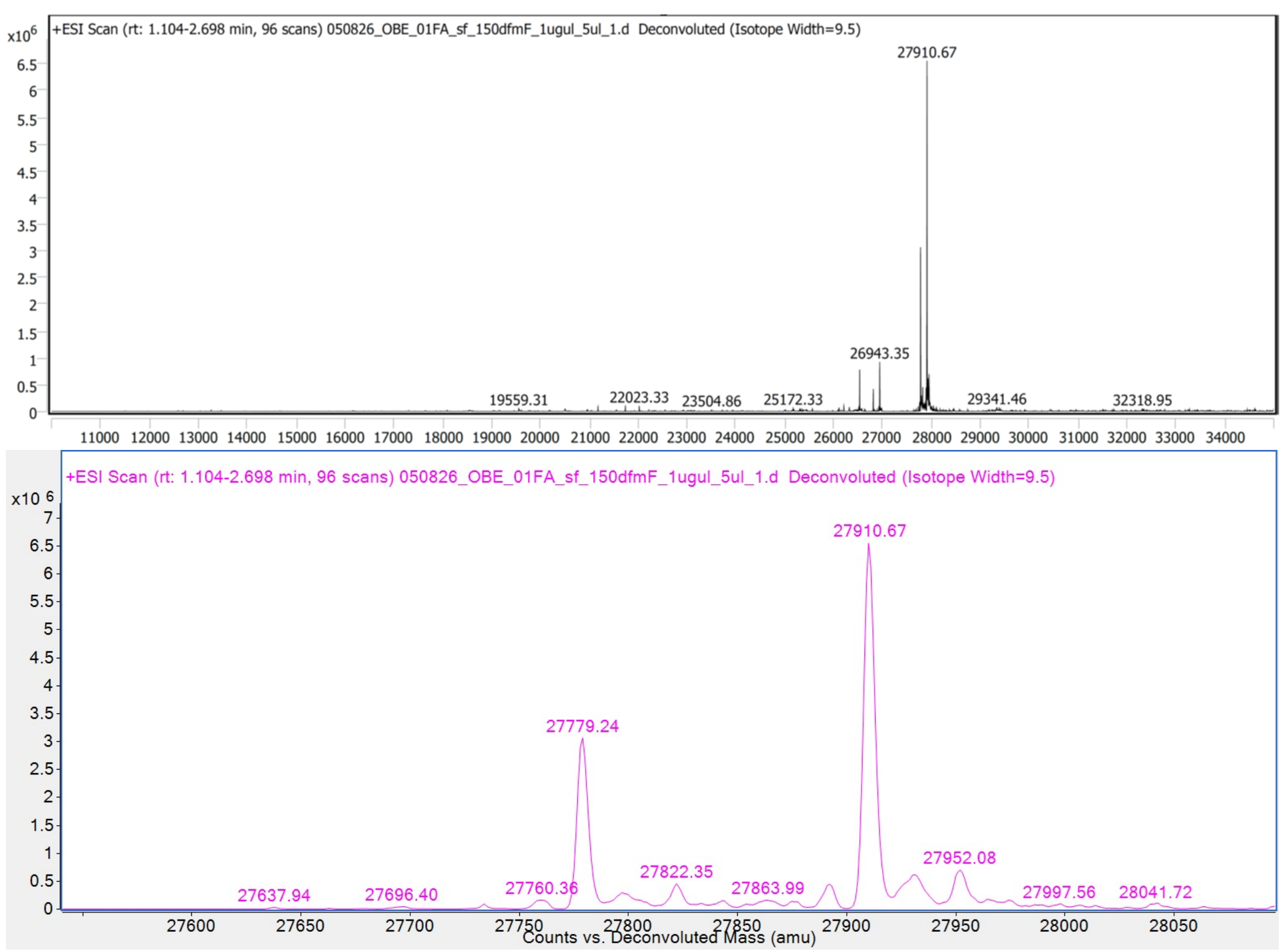

Figure S11. Intact mass spectrometric data of sfGFP-dfmF-150. Predicted 27910; Found 27911, and 27779 corresponding to the loss of one methionine residue.

MALDI.

Trypsin digest of sfGFP:

A purified sfGFP sample (20 µL) was combined with 40 mM NH_4HCO_3 aqueous solution (20 µL), 1% ProteaseMAX^{TM} Surfactant (Promega V2071, 1.00 µL), and 0.5 M DTT (0.50 µL). The resulting mixture was incubated at 65 °C for 20 min to denature the protein. The sample was digested using Trypsin Singles, Proteomics Grade Kit (Sigma-Aldrich, T7575) according to the manufacturer's directions. Briefly, trypsin solubilizing reagent (1.00 µL) was added to a trypsin singles vial. The denatured sample from the previous step (40.0 µL), trypsin reaction buffer (20.0 µL, 40 mM NH_4HCO_3 aq. and 9% v/v CH_3CN), and 1% ProteaseMAX^{TM} Surfactant (1.00 µL) were transferred to the vial. The digestion mixture was vortexed and centrifuged before overnight incubation at 37 °C. The samples were acidified with 1% v/v CF_3CO_2H aq. (1.00 µL). The samples were diluted 1:10 with 0.1% v/v CF_3CO_2H aq. and analyzed by MALDI-TOF spectrometry with α-cyano-4-hydroxycinnamic acid (CHCA) matrix.

sfGFP

```
MVSKGEELFT GVVPILVELD GDVNGHKFSV RGEGEGDATN GKLTLKFICT TGKLPVPWPT LVTTLTYGVQ CFSRYPDHMK
RHDFFKSAMP EGYVQERTIS FKDDGTYKTR AEVKFEGDTL VNRIELKGID FKEDGNILGH KLEYNFNSHN VYITADKQKN
GIKANFKIRH NVEDGSVQLA DHYQQNTPIG DGPVLLPDNH YLSTQSVLSK DPNEKRDHMV LLEFVTAAGI THGMDELYKG
SHHHHHH
```

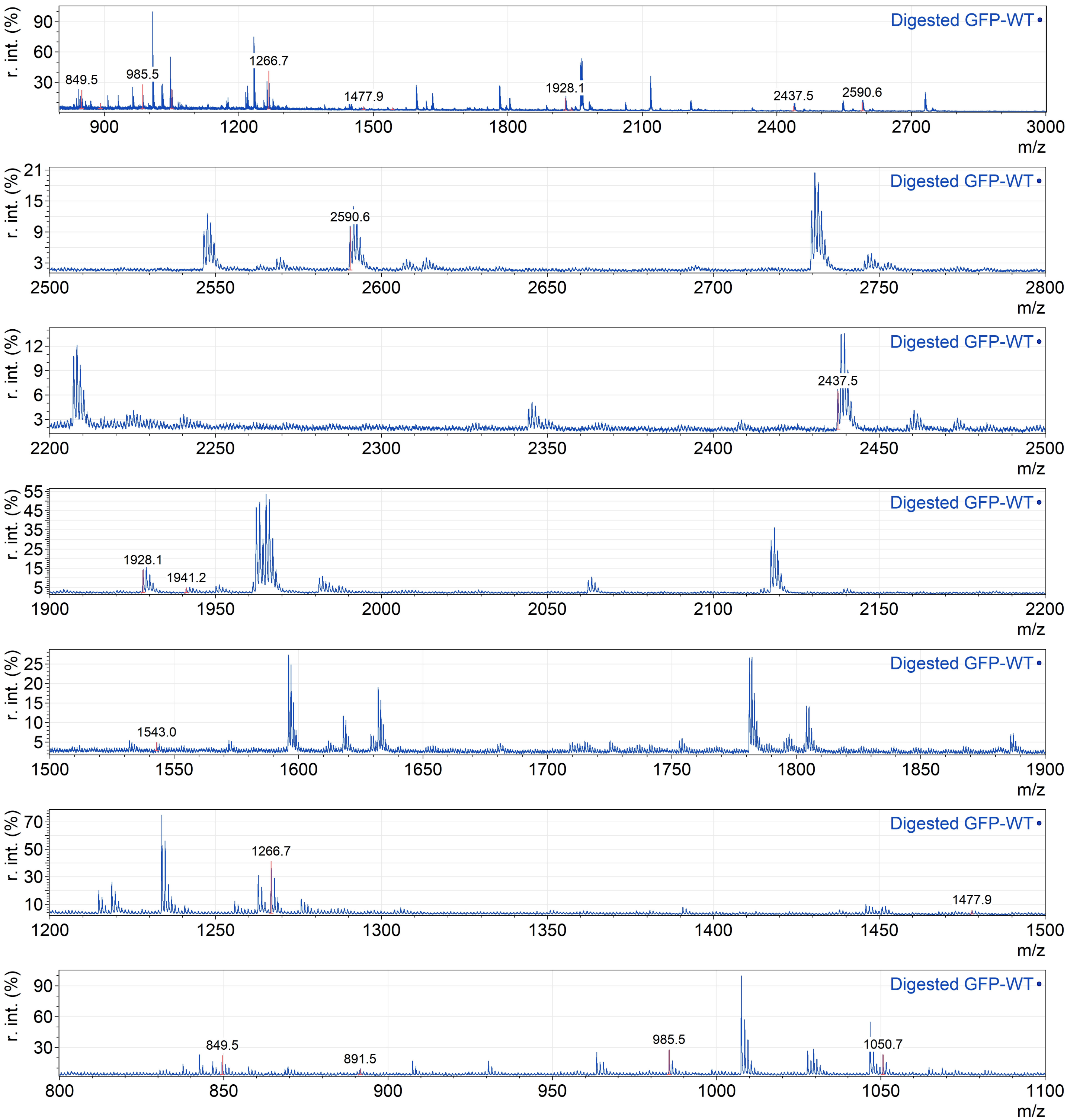

Protein	Obs. [M+H]$^+$ m/z	Calc. [M+H]$^+$ m/z	Position	Fragment sequence
sfGFP	2590.6	2590.3	217-239	DHMVLLEFVTAAGITHGMDELYK
sfGFP	2437.5	2437.3	5-27	GEELFTGVVPILVELDGDVNGHK
sfGFP	1941.2	1940.9	82-97	HDFFKSAMPEGYVQER
sfGFP	1928.1	1927.9	142-157	LEYNFNSH**N**VYITADK
sfGFP	1543.0	1542.8	128.141	GIDFKEDGNILGHK
sfGFP	1477.9	1477.8	111-123	AEVKFEGDTLVNR
sfGFP	1266.7	1266.6	87-97	SAMPEGYVQER
sfGFP	1050.7	1050.5	115-123	FEGDTLVNR
sfGFP	985.5	985.4	240-247	GSHHHHHH
sfGFP	891.5	891.5	160-167	NGIKANFK
sfGFP	849.5	849.4	81-86	RHDFFK

Figure S12. MALDI MS data of digested sfGFP showing m/z from 800 to 3000. Identified fragments are indicated.

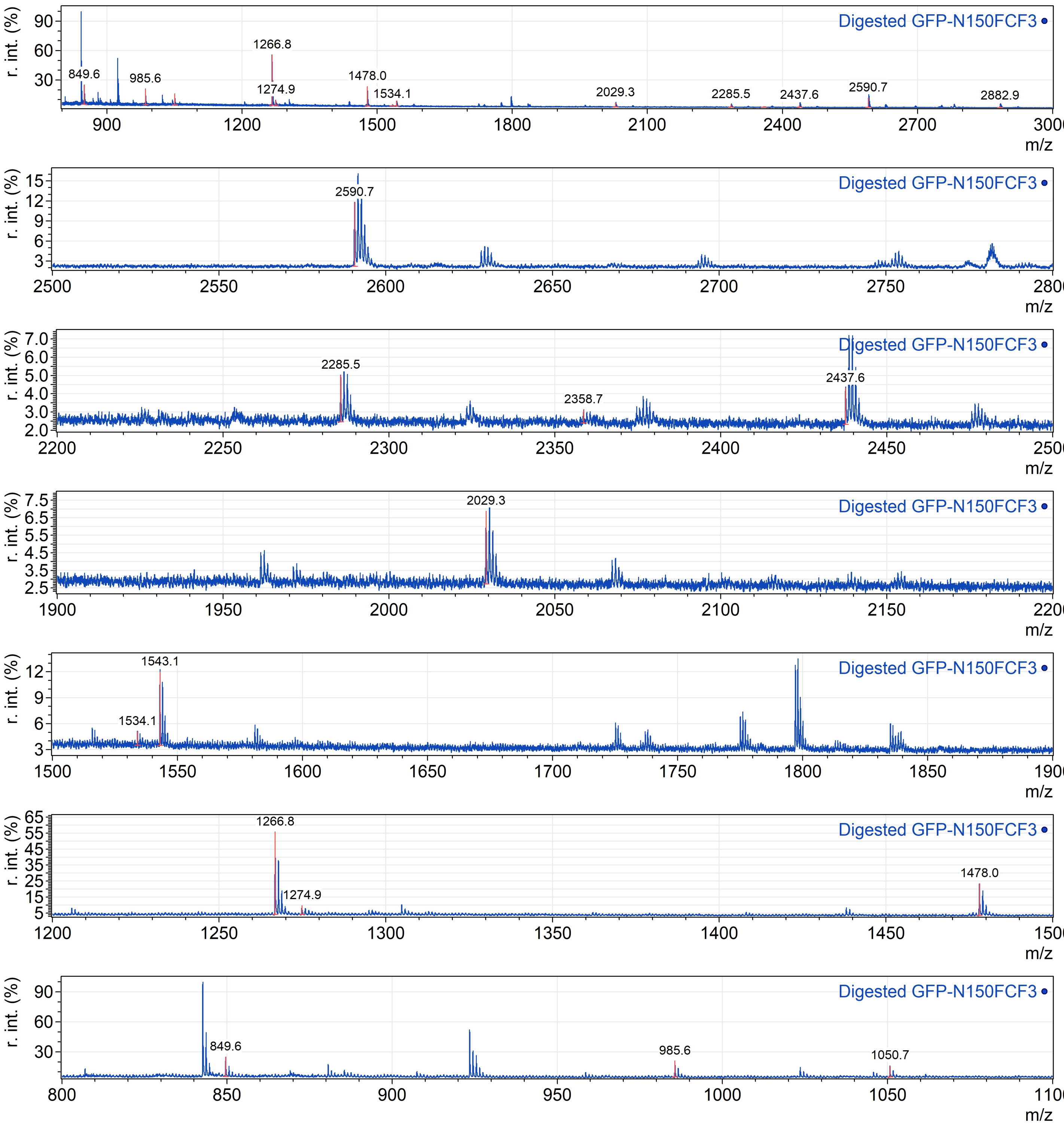

Protein	Obs. $[M+H]^+$ m/z	Calc. $[M+H]^+$ m/z	Position	Fragment sequence
sfGFP-tfmF-150	2590.7	2590.3	217-239	DHMVLLEFVTAAGITHGMDELYK
sfGFP-tfmF-150	2437.6	2437.3	5-27	GEELFTGVVPILVELDGDVNGHK
sfGFP-tfmF-150	2285.5	2285.1	142-159	LEYNFNSH**F(CF_3)**VYITADKQK
sfGFP-tfmF-150	2029.3	2028.9	142-157	LEYNFNSH**F(CF_3)**VYITADK
sfGFP-tfmF-150	1543.1	1542.8	128-141	GIDFKEDGNILGHK
sfGFP-tfmF-150	1534.1	1533.8	115-127	FEGDTLVNRIELK
sfGFP-tfmF-150	1478.0	1477.8	111-123	AEVKFEGDTLVNR
sfGFP-tfmF-150	1274.9	1274.6	98-108	TISFKDDGTYK
sfGFP-tfmF-150	1266.8	1266.6	87-97	SAMPEGYVQER
sfGFP-tfmF-150	1050.7	1050.5	115-123	FEGDTLVNR
sfGFP-tfmF-150	985.6	985.4	240-247	GSHHHHHH
sfGFP-tfmF-150	849.5	849.4	81-86	RHDFFK

Figure S13. MALDI MS data of digested sfGFP-tfmF-150 showing m/z from 800 to 3000. Identified fragments are indicated.

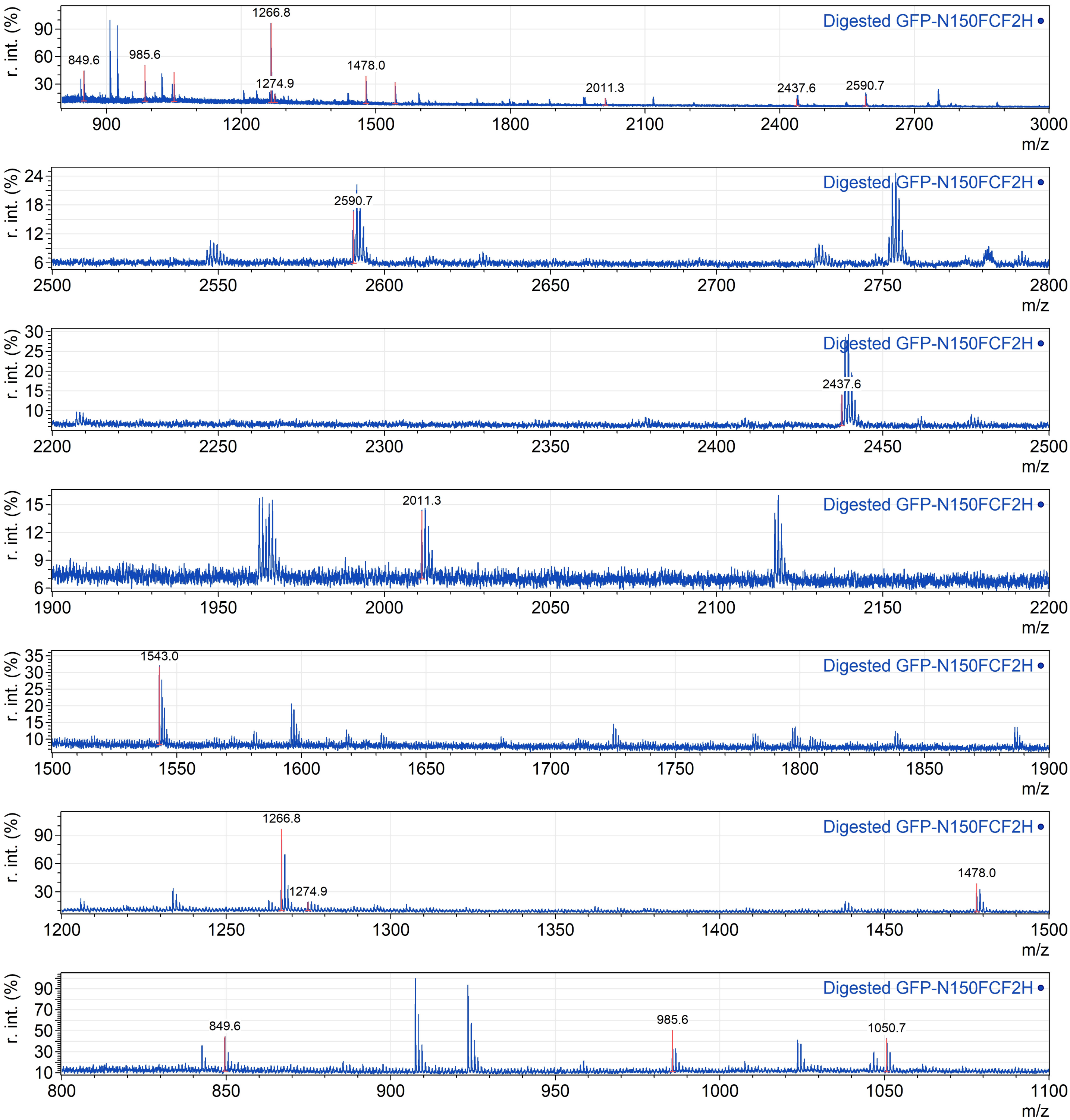

Protein	Obs. $[M+H]^+$ m/z	Calc. $[M+H]^+$ m/z	Position	Fragment sequence
sfGFP-dfmF-150	2590.7	2590.3	217-239	DHMVLLEFVTAAGITHGMDELYK
sfGFP-dfmF-150	2437.6	2437.3	5-27	GEELFTGVVPILVELDGDVNGHK
sfGFP-dfmF-150	2011.3	2010.9	142-157	LEYNFNSH**F(CF$_2$H)**VYITADK
sfGFP-dfmF-150	1543.0	1542.8	128-141	GIDFKEDGNILGHK
sfGFP-dfmF-150	1478.0	1477.8	111-123	AEVKFEGDTLVNR
sfGFP-dfmF-150	1274.9	1274.6	98-108	TISFKDDGTYK
sfGFP-dfmF-150	1266.8	1266.6	87-97	SAMPEGYVQER
sfGFP-dfmF-150	1050.7	1050.5	115-123	FEGDTLVNR
sfGFP-dfmF-150	985.6	985.4	240-247	GSHHHHHH
sfGFP-dfmF-150	849.6	849.4	81-86	RHDFFK

Figure S14. MALDI MS data of digested sfGFP-dfmF-150 showing m/z from 800 to 3000. Identified fragments are indicated.

Trypsin Digest of Carbonic Anhydrase

Stock solutions of wild-type and mutant CA protein samples were diluted to a concentration of 30 μM with tris buffer (40 mM, pH 10.5). A portion of each protein sample was then digested with a Trypsin Singles, Proteomics Grade Kit (Sigma-Aldrich T7575). For each sample, an aliquot of trypsin solubilization reagent (1.00 μL) was added to a trypsin singles vial, followed by the protein sample (10.0 μL) and trypsin reaction buffer (49.0 μL, 40 mM NH_4HCO_3 aq. and 9% v/v CH_3CN). The reaction mixtures were incubated overnight at 37 °C. The samples were then acidified with 10% v/v CF_3CO_2H aq. (1.60 μL) and diluted 1:10 with CH_3CN and analyzed by MALDI-TOF spectrometry using α-cyano-4-hydroxycinnamic acid (CHCA) as a matrix.

CA

```
MAHHWGYGKH NGPEHWHKDF PIAKGERQSP VDIDTHTAKY DPSLKPLSVS YDQATSLRIL NNGHTFNVEF DDSQDKAVLK
GGPLDGTYRL IQFHFHWGSH DGQGSEHTVD KKKYAAELHL VHWNTKYGDF GKAVQQPDGL AVLGIFLKVG SANPGLQKVV
DVLDSIKTKG KSADFTNFDP RGLLPESLDY WTYPGSLTTP PLLECVTWIV LKEPISVSSE QVSKFRKLNF NGEGEPEEPM
VDNWRPTQPL KNRQIKASFK HHHHHH
```

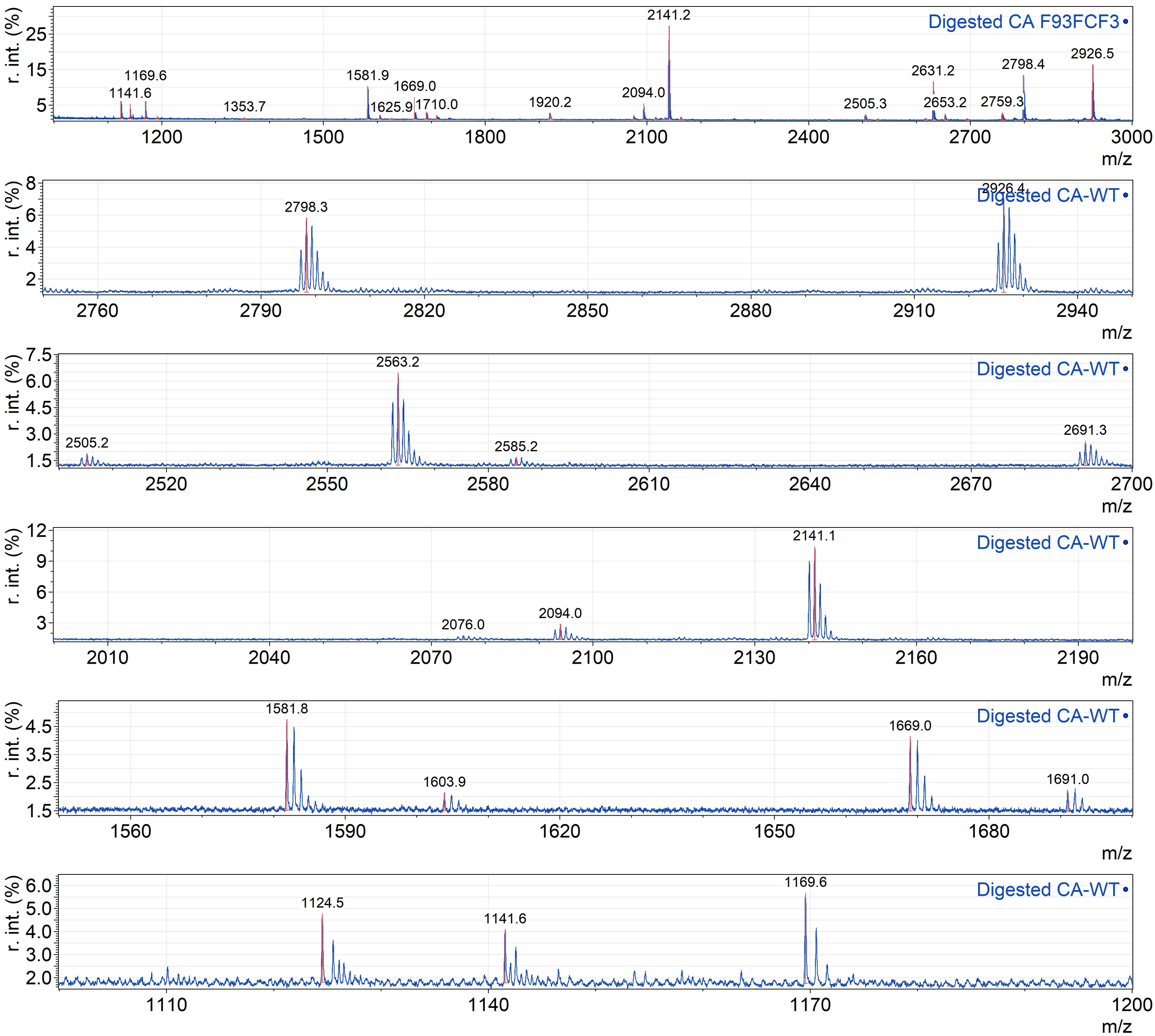

Protein	Obs. $[M+H]^+$ m/z	Calc. $[M+H]^+$ m/z	Position	Fragment sequence
Carbonic anhydrase	2926.4	2926.4	227-251	KLNFNGEGEPEEPMVDNWRPTQPLK
Carbonic anhydrase	2798.3	2798.3	228-251	LNFNGEGEPEEPMVDNWRPTQPLK
Carbonic anhydrase	2691.3	2691.3	90-112	LIQ**F**HFHWGSHDGQGSEHTVDKK
Carbonic anhydrase	2563.2	2563.2	90-111	LIQ**F**HFHWGSHDGQGSEHTVDK
Carbonic anhydrase	2505.2	2505.2	59-80	ILNNGHTFNVEFDDSQDKAVLK
Carbonic anhydrase	2141.1	2141.1	40-58	YDPSLKPLSVSYDQATSLR
Carbonic anhydrase	2094.0	2094.0	59-76	ILNNGHTFNVEFDDSQDK
Carbonic anhydrase	1669.0	1669.0	133-148	AVQQPDGLAVLGIFLK
Carbonic anhydrase	1581.8	1581.8	114-126	YAAELHLVHWNTK
Carbonic anhydrase	1169.6	1169.5	172-181	SADFTNFDPR
Carbonic anhydrase	1141.8	1141.5	10-18	HNGPEHWHK

Figure S15. MALDI MS data of digested CA wild-type showing m/z from 1000 to 3000. Identified fragments are indicated.

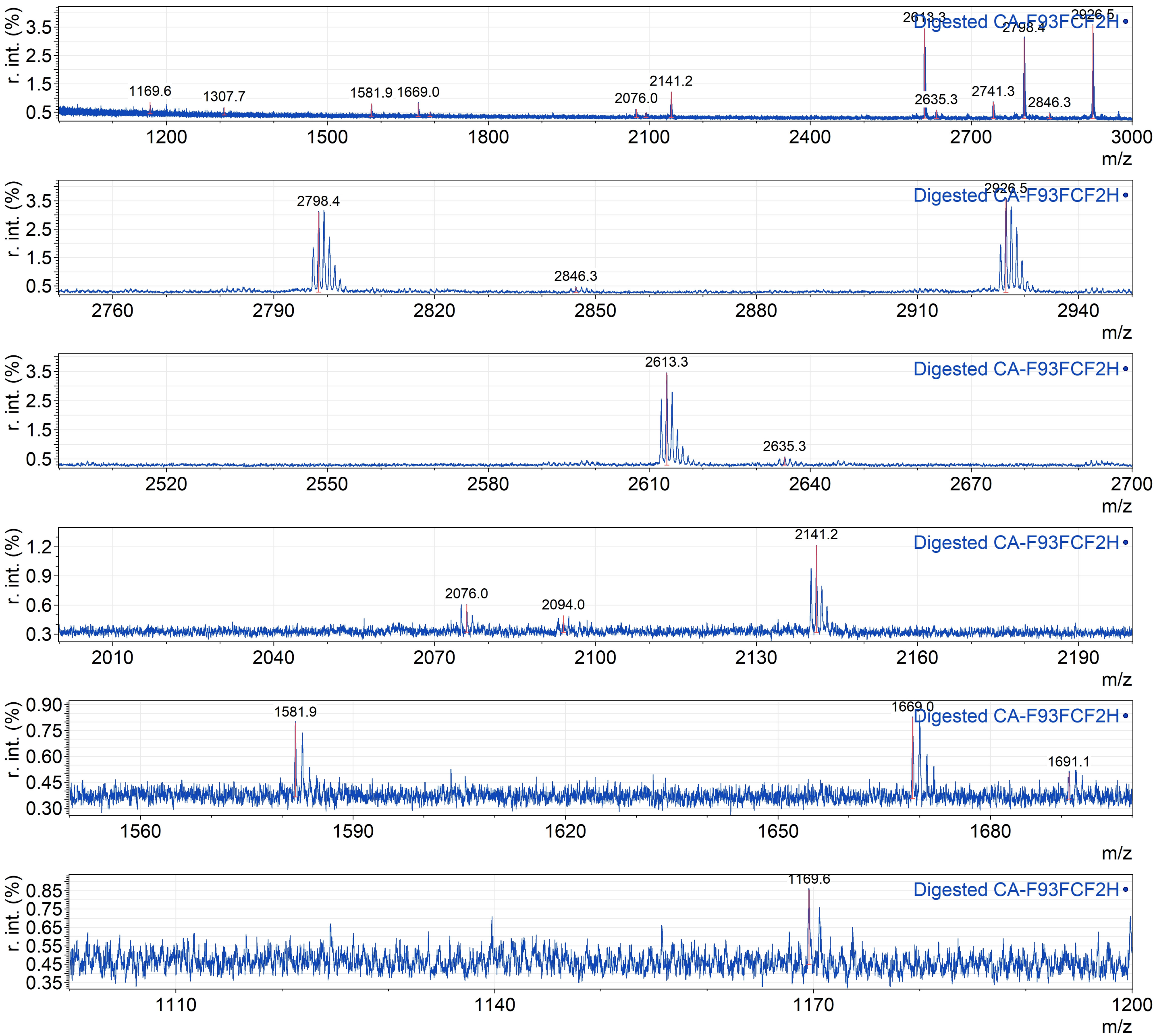

Protein	Obs. $[M+H]^+$ m/z	Calc. $[M+H]^+$ m/z	Position	Fragment sequence
Carbonic anhydrase	2926.5	2926.4	227-251	KLNFNGEGEPEEPMVDNWRPTQPLK
Carbonic anhydrase	2798.4	2798.3	228-251	LNFNGEGEPEEPMVDNWRPTQPLK
Carbonic anhydrase	2741.3	2741.3	90-112	LIQ**F(CF_2H)**HFHWGSHDGQGSEHTVDKK
Carbonic anhydrase	2613.3	2613.2	90-111	LIQ **F(CF_2H)**HFHWGSHDGQGSEHTVDK
Carbonic anhydrase	2141.2	2141.1	40-58	YDPSLKPLSVSYDQATSLR
Carbonic anhydrase	2094.0	2094.0	59-76	ILNNGHTFNVEFDDSQDK
Carbonic anhydrase	1669.0	1669.0	133-148	AVQQPDGLAVLGIFLK
Carbonic anhydrase	1581.9	1581.8	114-126	YAAELHLVHWNTK
Carbonic anhydrase	1169.6	1169.5	172-181	SADFTNFDPR

Figure S16. MALDI MS data of digested CA-F93F(CF_2H) showing m/z from 1000 to 3000. Identified fragments are indicated.

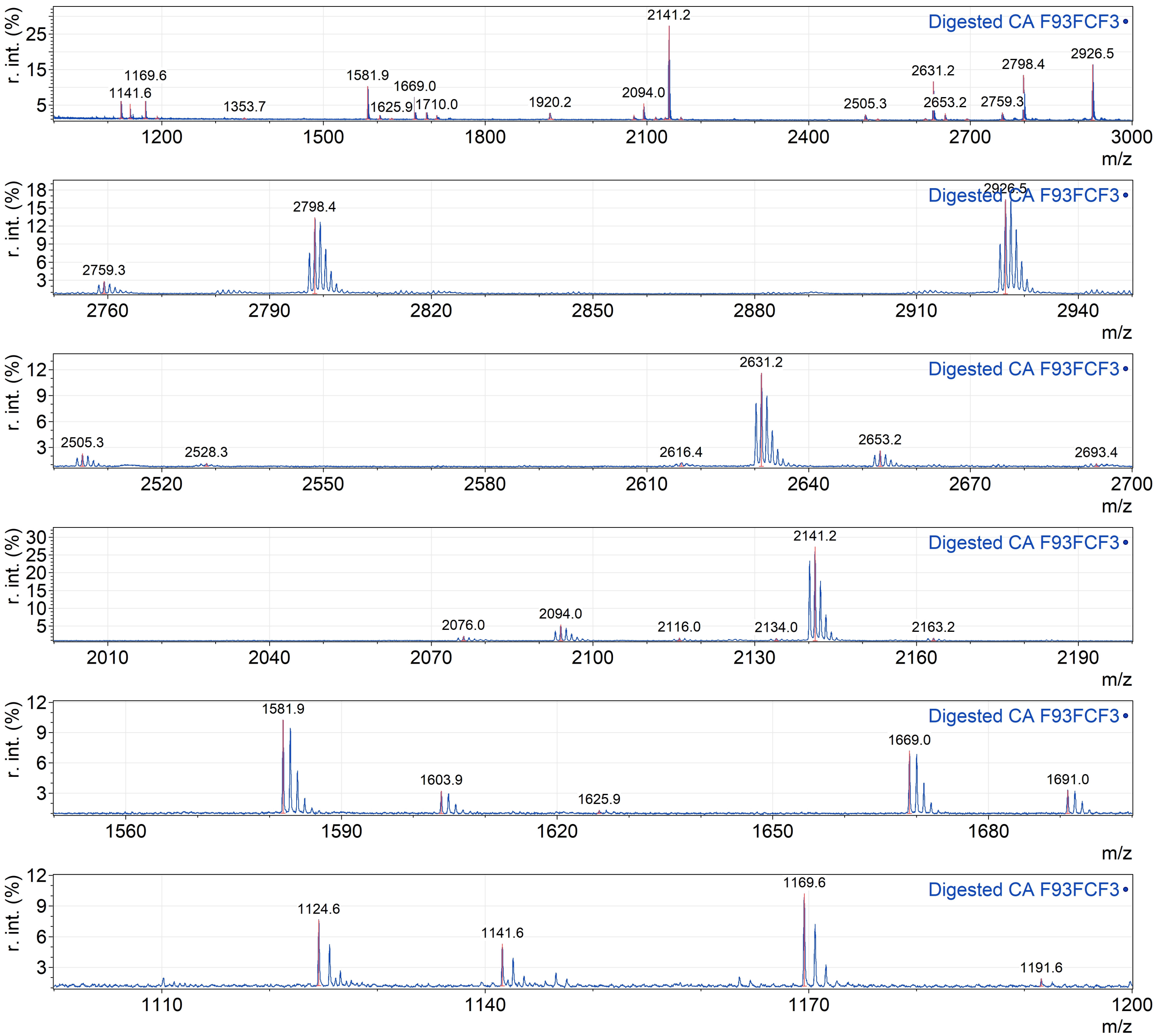

Protein	Obs. $[M+H]^+$ m/z	Calc. $[M+H]^+$ m/z	Position	Fragment sequence
Carbonic anhydrase	2926.5	2926.4	227-251	KLNFNGEGEPEEPMVDNWRPTQPLK
Carbonic anhydrase	2798.4	2798.3	228-251	LNFNGEGEPEEPMVDNWRPTQPLK
Carbonic anhydrase	2798.4	2798.3	90-112	LIQ**F(CF_3)**HFHWGSHDGQGSEHTVDKK
Carbonic anhydrase	2631.2	2631.2	90-111	LIQ **F(CF_3)**HFHWGSHDGQGSEHTVDK
Carbonic anhydrase	2141.1	2141.2	40-58	YDPSLKPLSVSYDQATSLR
Carbonic anhydrase	2094.0	2094.0	59-76	ILNNGHTFNVEFDDSQDK
Carbonic anhydrase	1710.0	1709.9	113-126	KYAAELHLVHWNTK
Carbonic anhydrase	1669.0	1669.0	133-148	AVQQPDGLAVLGIFLK
Carbonic anhydrase	1581.8	1581.9	114-126	YAAELHLVHWNTK
Carbonic anhydrase	1354.7	1354.6	170-181	GKSADFTNFDPR
Carbonic anhydrase	1169.6	1169.6	172-181	SADFTNFDPR
Carbonic anhydrase	1141.6	1141.5	10-18	HNGPEHWHK

Figure S17. MALDI MS data of digested CA-F93F(CF_3) showing m/z from 1000 to 3000. Identified fragments are indicated.

NMR Spectra.

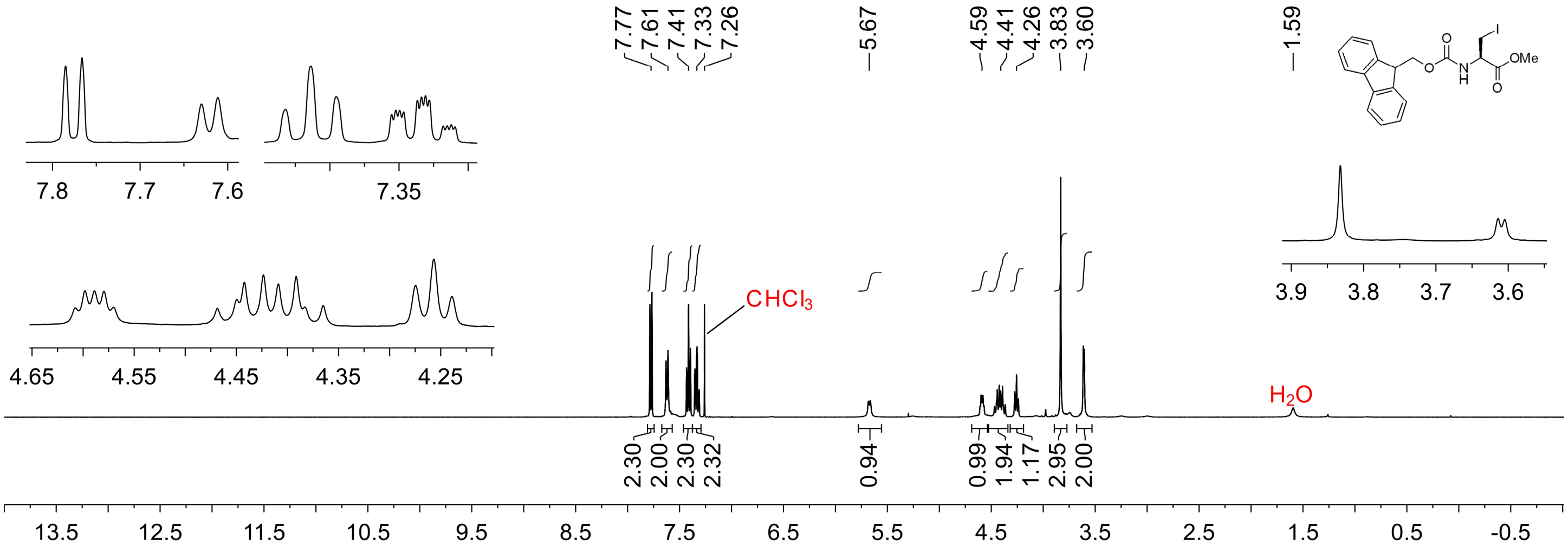

Figure S18. ^{1}H NMR spectrum of *N*-Fmoc-3-iodo-L-alanine methyl ester.

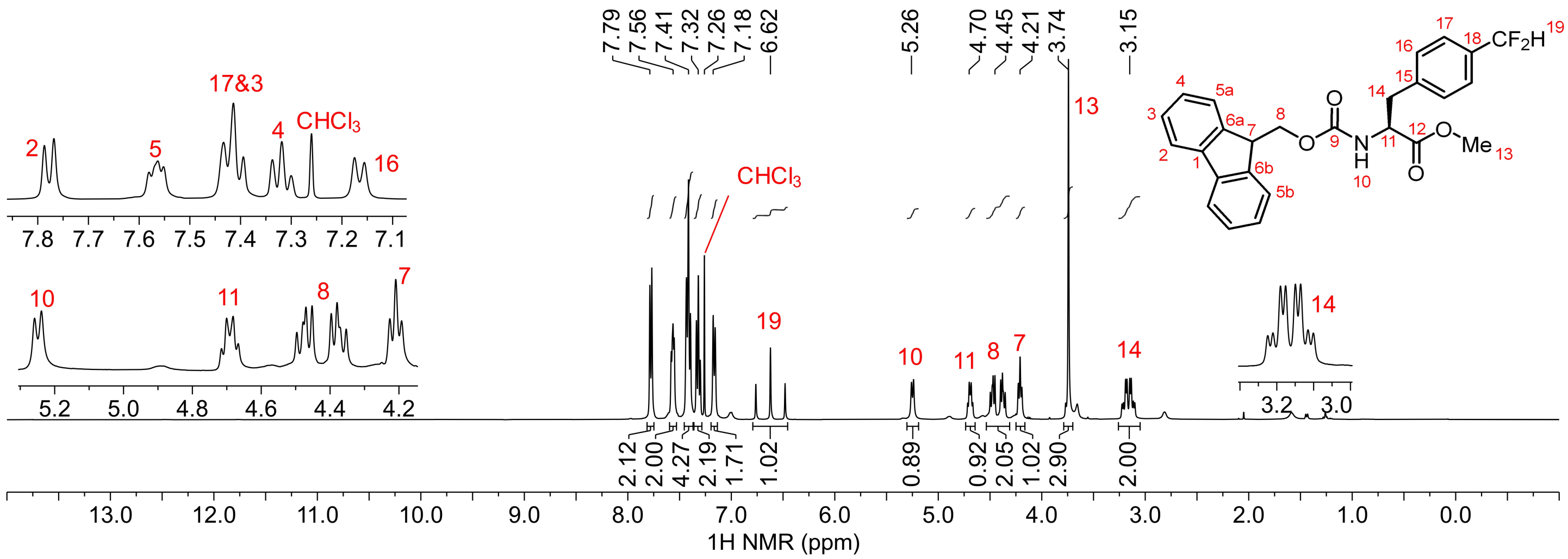

Figure S19. ^{1}H NMR spectrum of *N*-Fmoc-4-difluoromethyl-L-phenylalanine methyl ester.

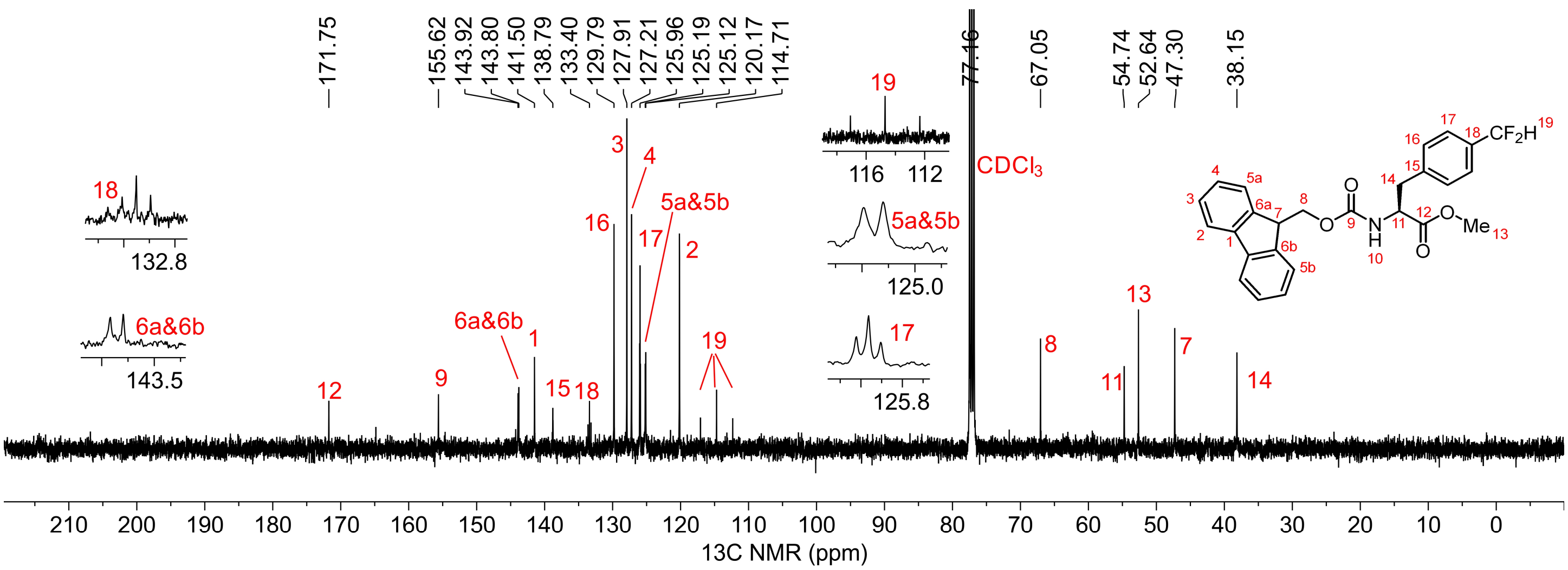

Figure S20. ^{13}C{^{1}H} NMR spectrum of *N*-Fmoc-4-difluoromethyl-L-phenylalanine methyl ester.

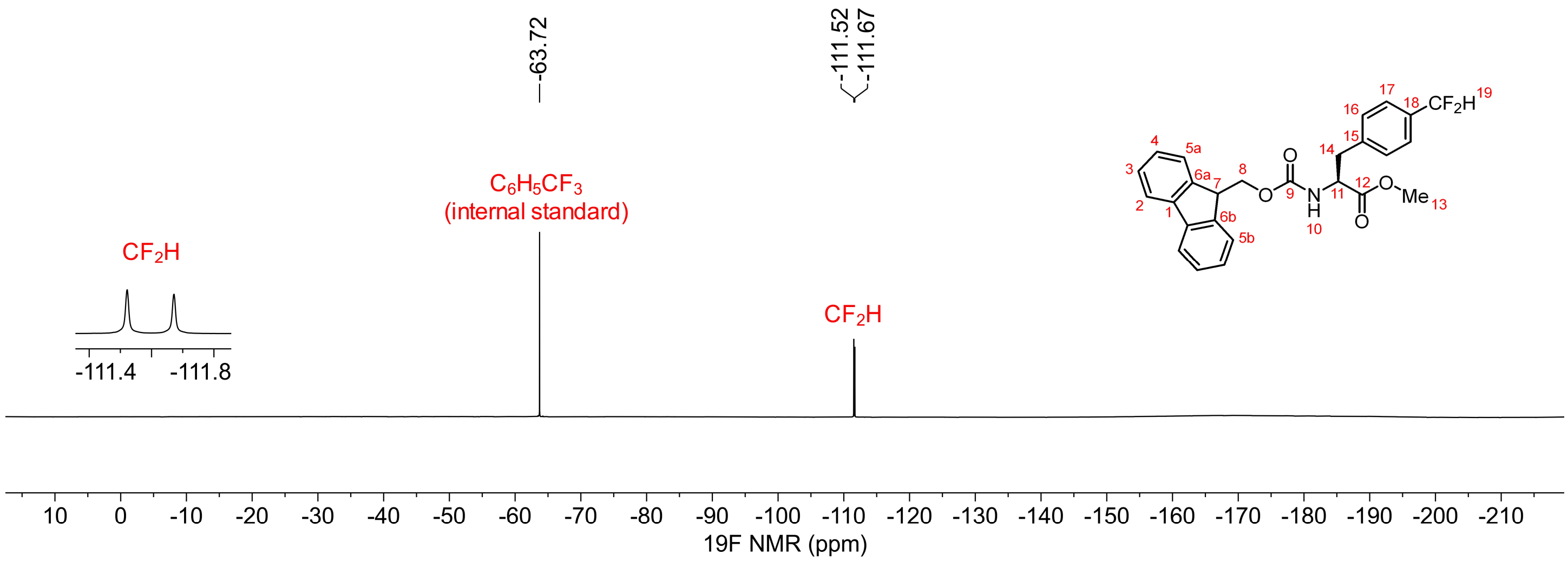

Figure S21. ^{19}F NMR spectrum of *N*-Fmoc-4-difluoromethyl-L-phenylalanine methyl ester.

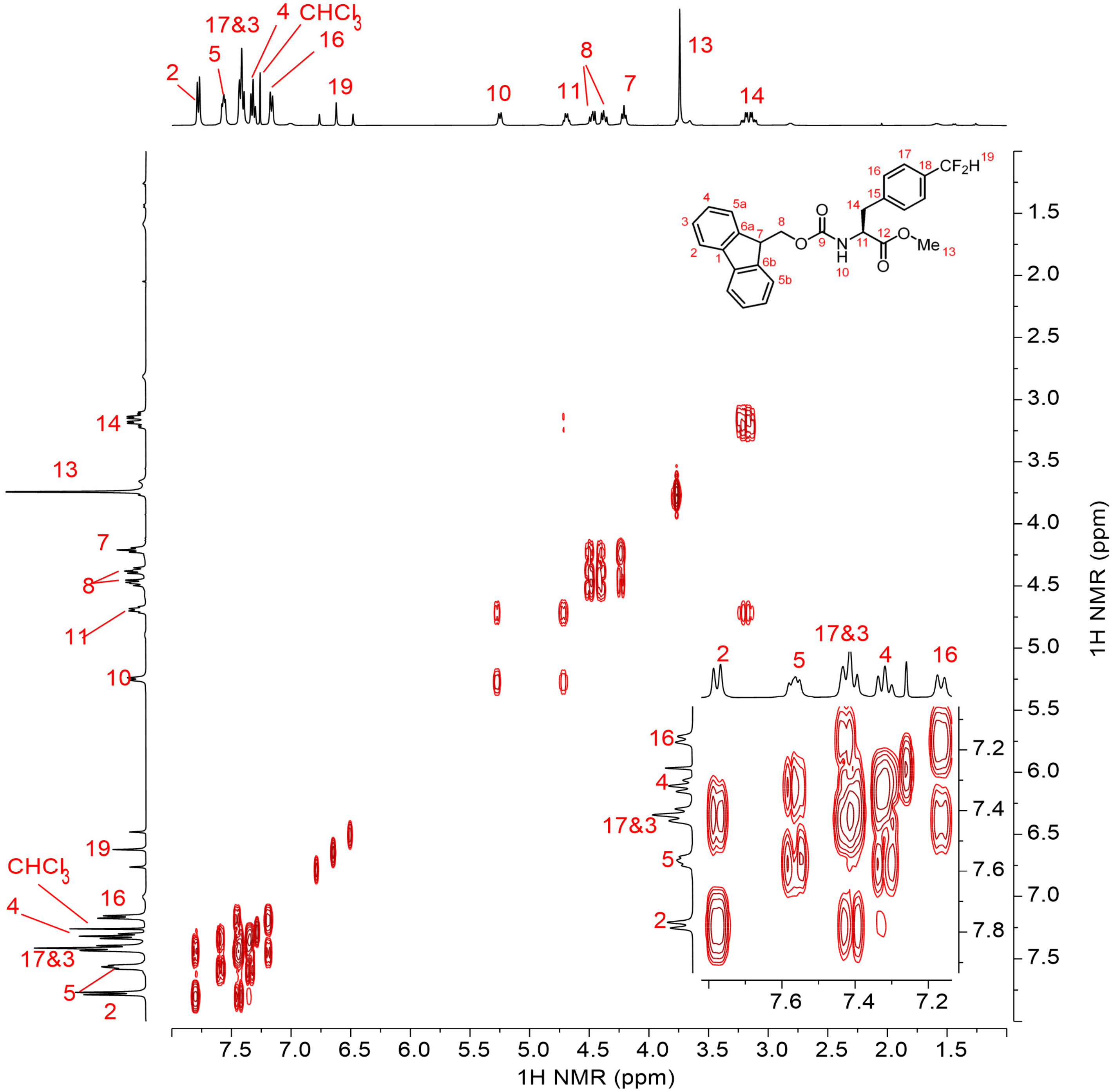

Figure S22. ^{1}H-^{1}H COSY spectrum of *N*-Fmoc-4-difluoromethyl-L-phenylalanine methyl ester.

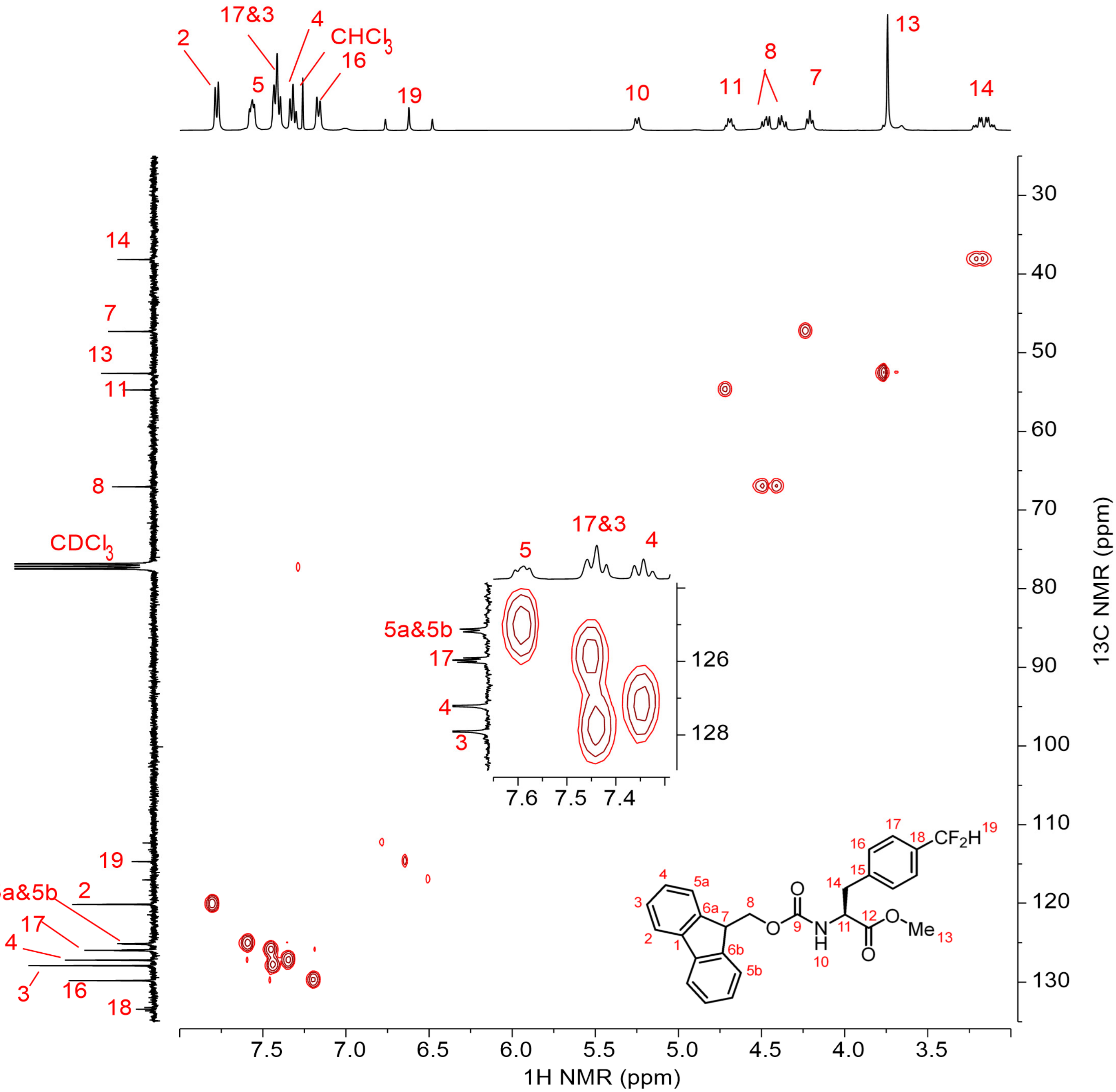

Figure S23. ^{1}H-^{13}C HSQC spectrum of *N*-Fmoc-4-difluoromethyl-L-phenylalanine methyl ester.

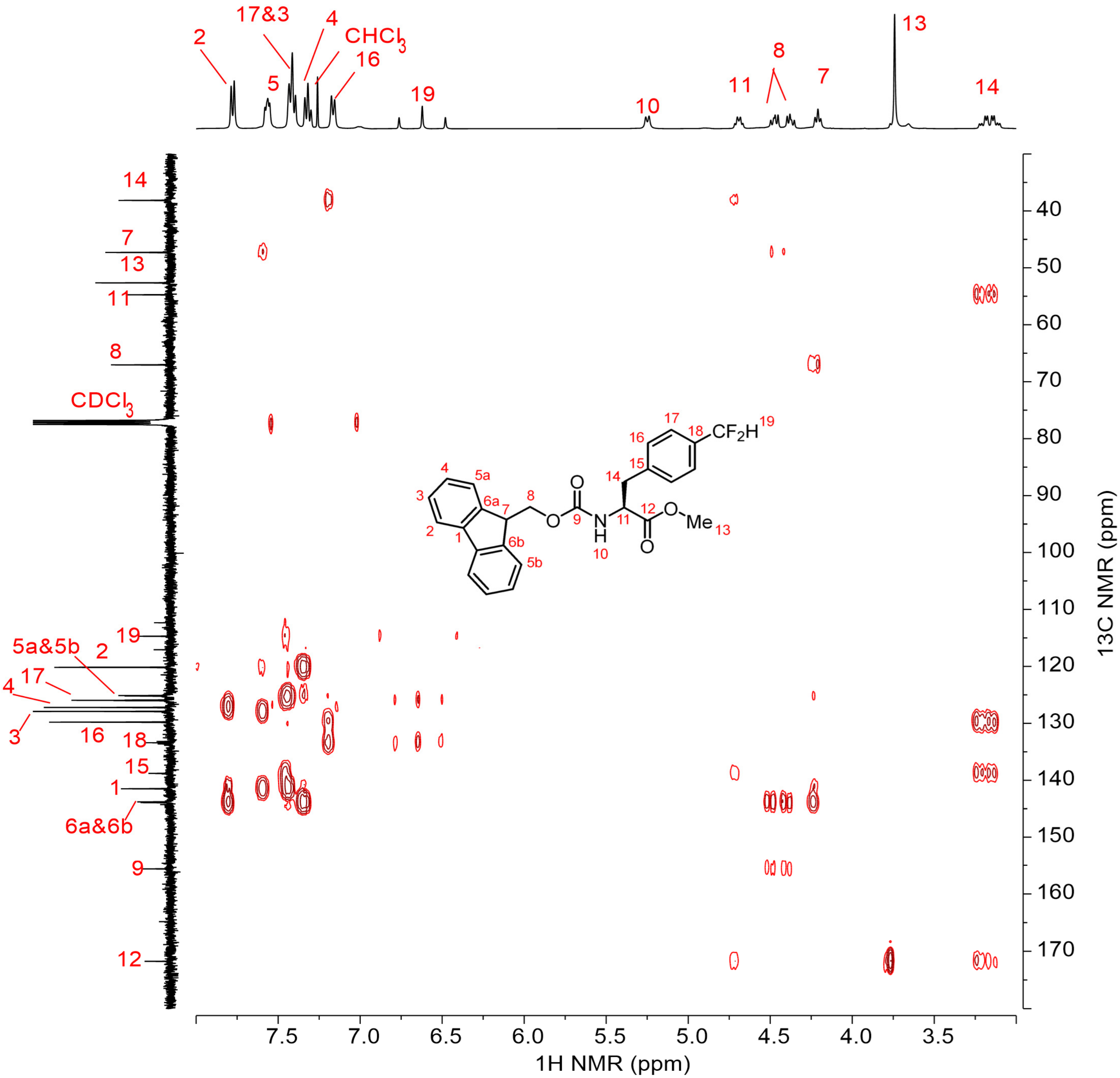

Figure S24. ^{1}H-^{13}C HMBC spectrum of *N*-Fmoc-4-difluoromethyl-L-phenylalanine methyl ester.

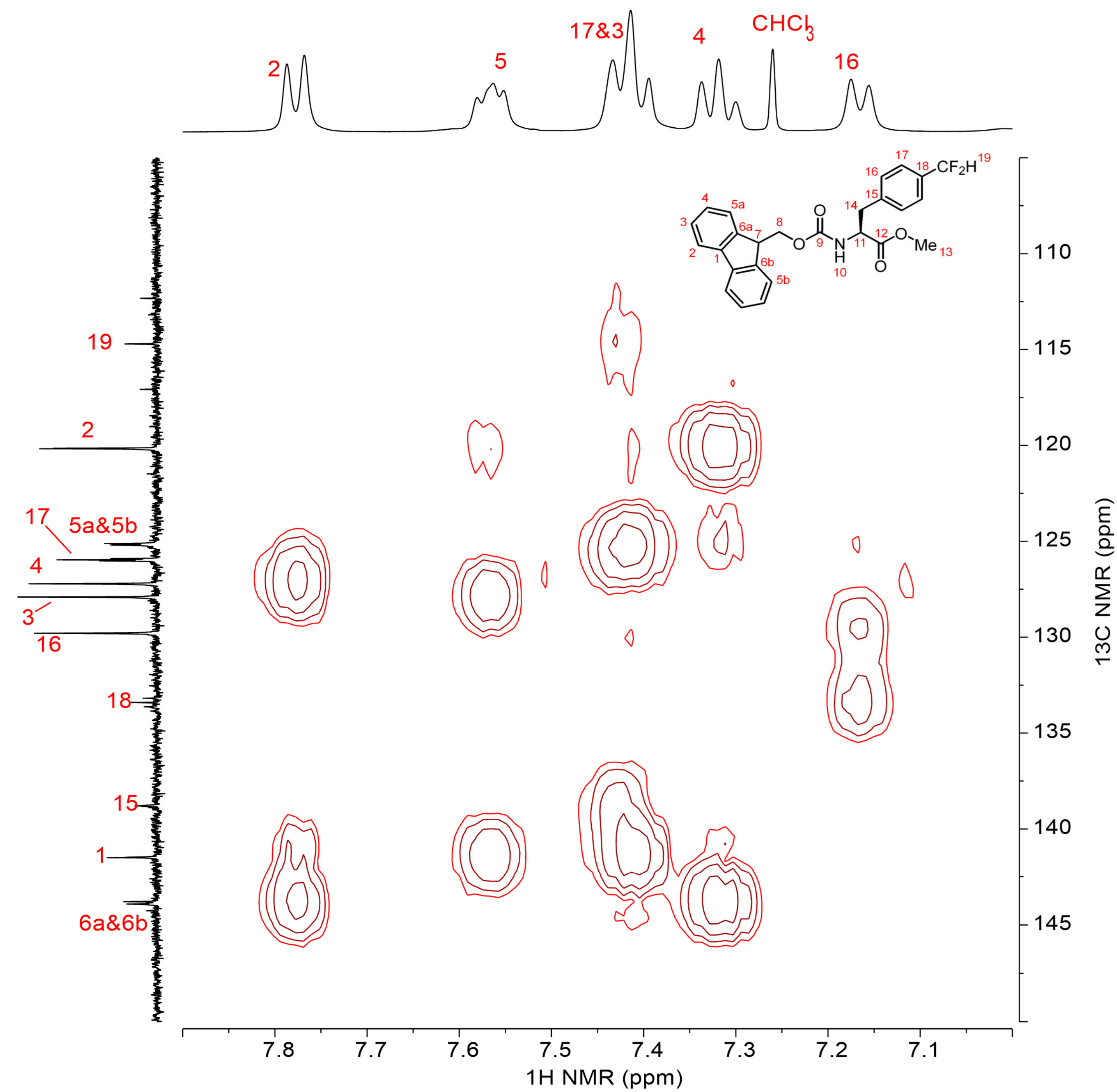

Figure S25. Expansion of ^{1}H-^{13}C HMBC spectrum of *N*-Fmoc-4-difluoromethyl-L-phenylalanine methyl ester (7.0–7.9 ppm for ^{1}H and 105–150 ppm for ^{13}C).

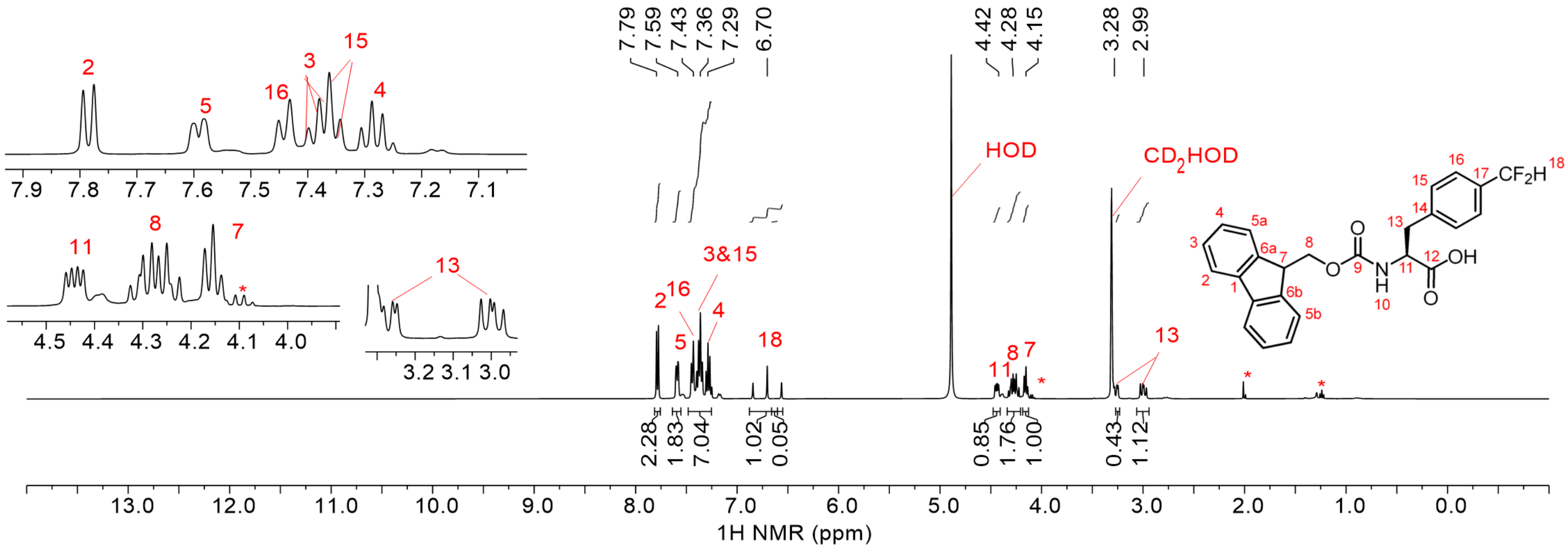

Figure S26. ^{1}H NMR spectrum of *N*-Fmoc-4-difluoromethyl-L-phenylalanine.

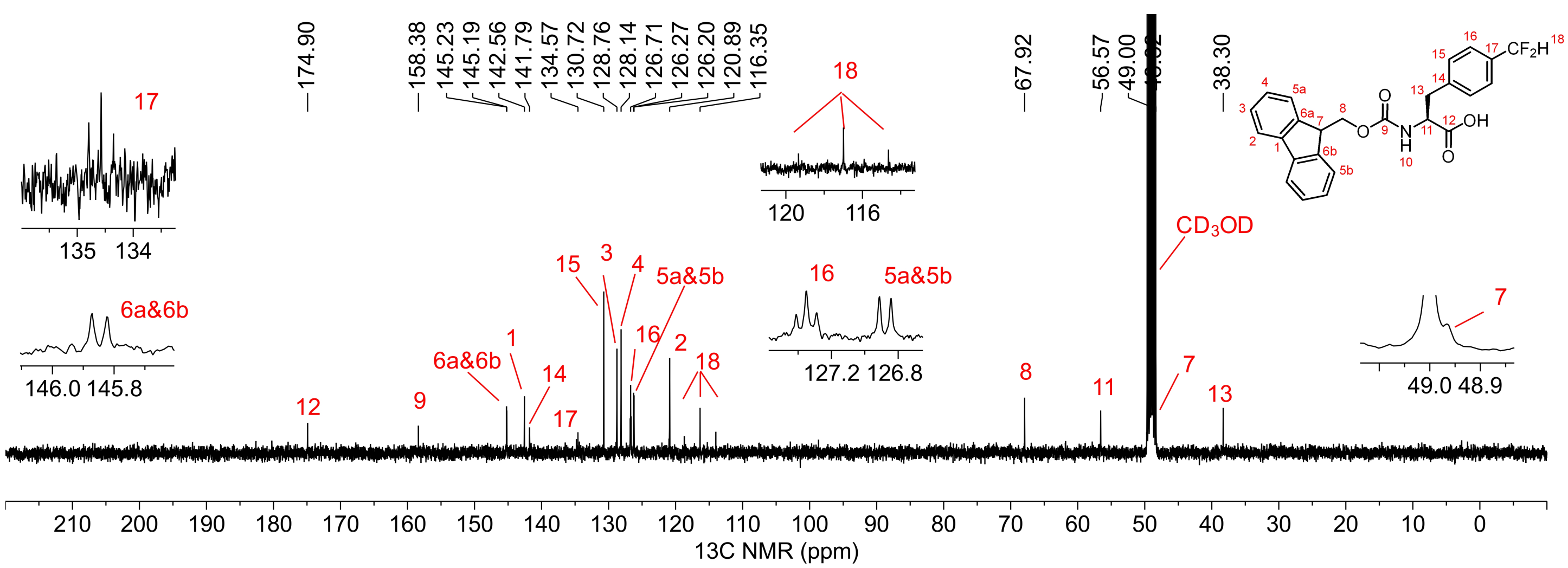

Figure S27. ^{13}C{^{1}H} NMR spectrum of *N*-Fmoc-4-difluoromethyl-L-phenylalanine.

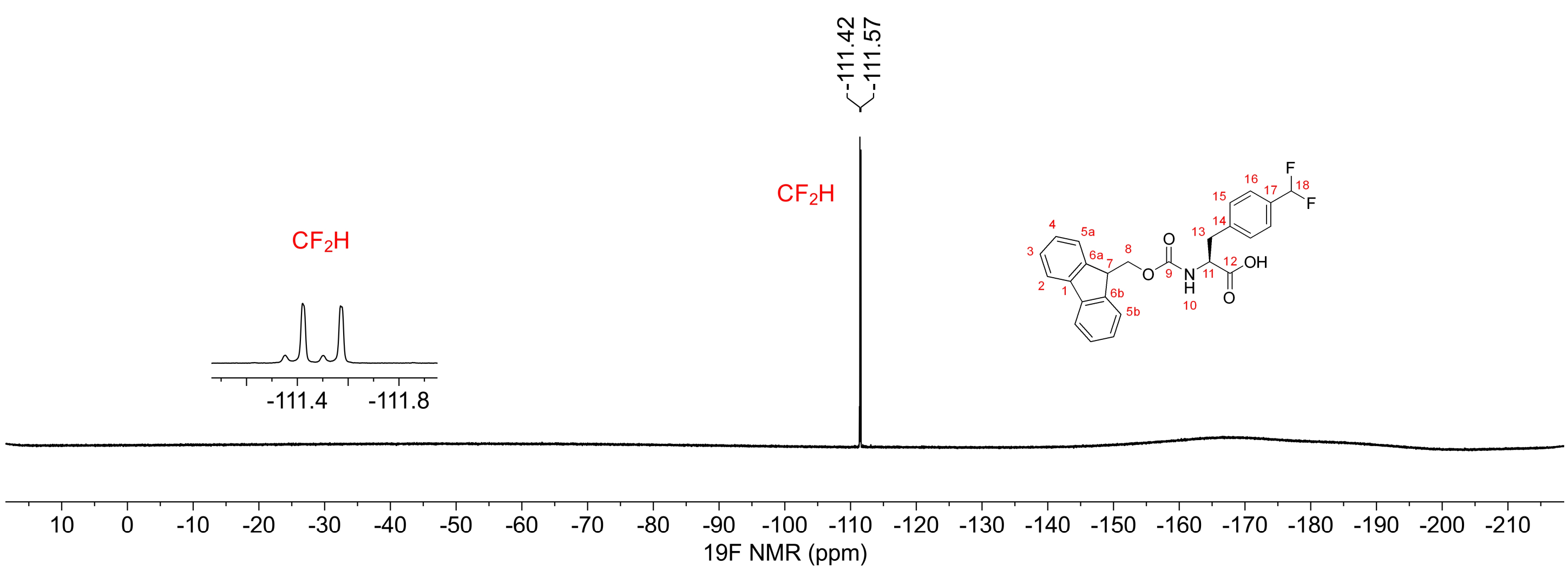

Figure S28. ^{19}F NMR spectrum of *N*-Fmoc-4-difluoromethyl-L-phenylalanine.

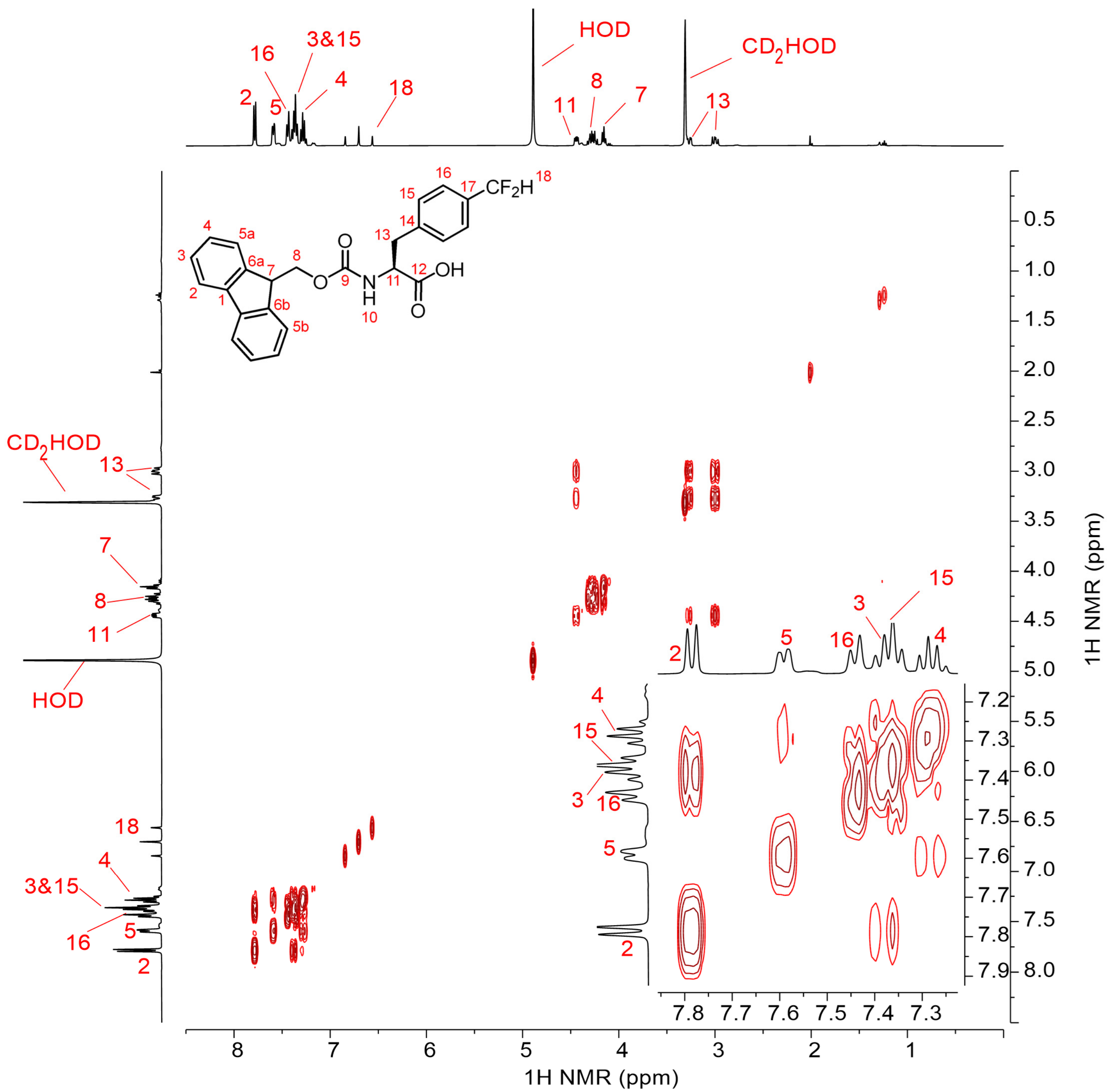

Figure S29. ^{1}H-^{1}H COSY spectrum of *N*-Fmoc-4-difluoromethyl-L-phenylalanine.

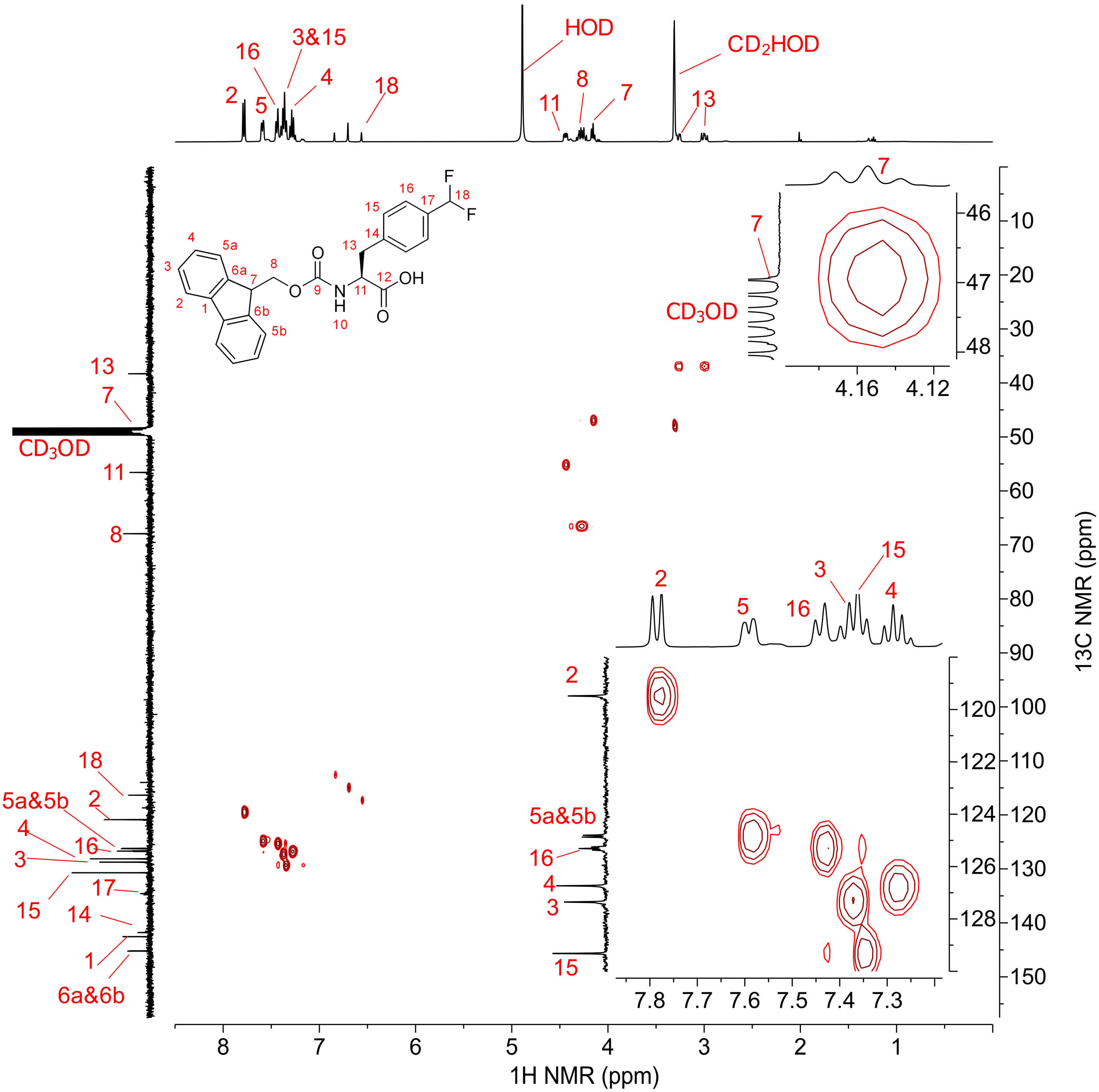

Figure S30. ^{1}H-^{13}C HSQC spectrum of *N*-Fmoc-4-difluoromethyl-L-phenylalanine.

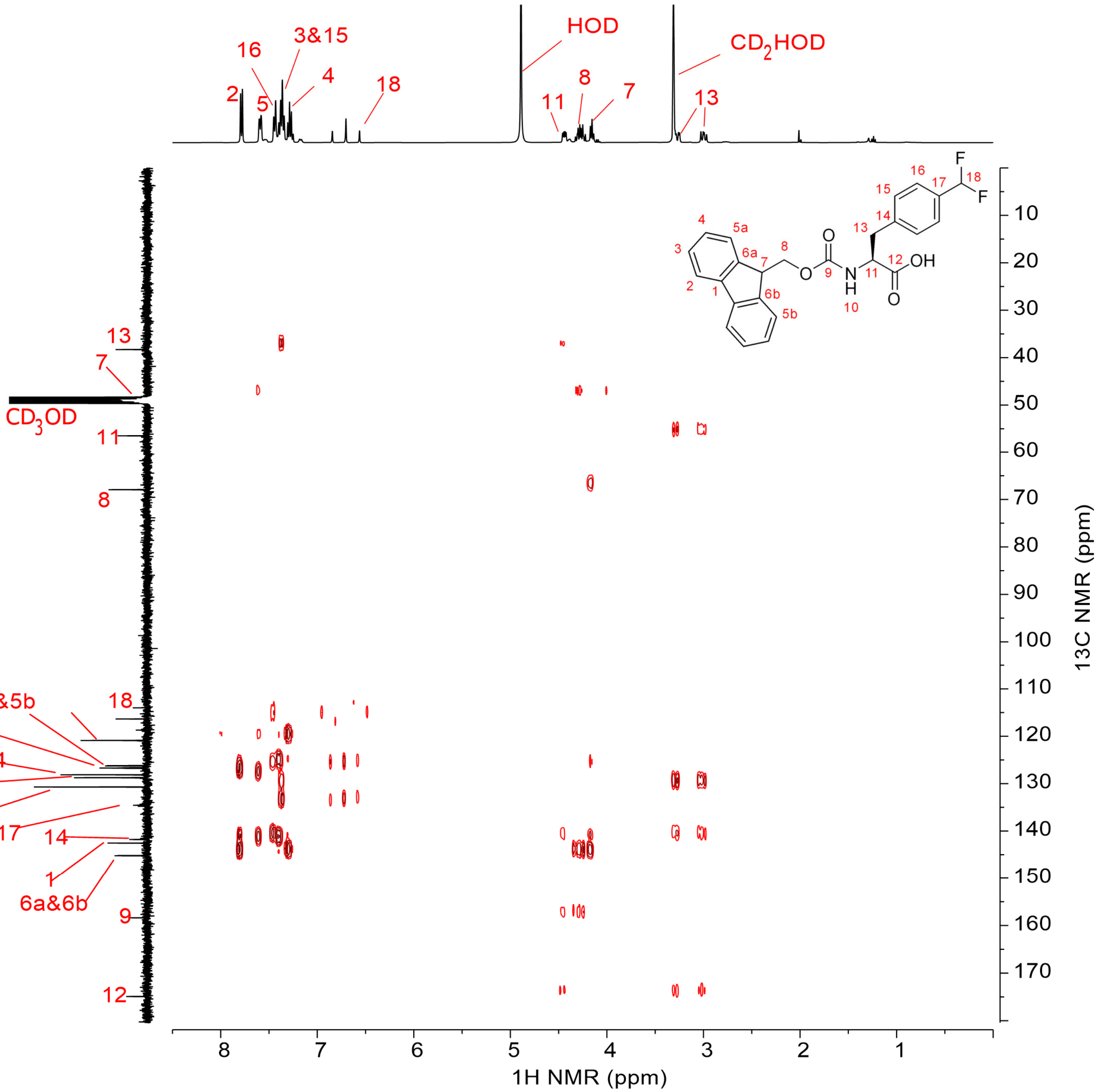

Figure S31. ^{1}H-^{13}C HMBC spectrum of *N*-Fmoc-4-difluoromethyl-L-phenylalanine.

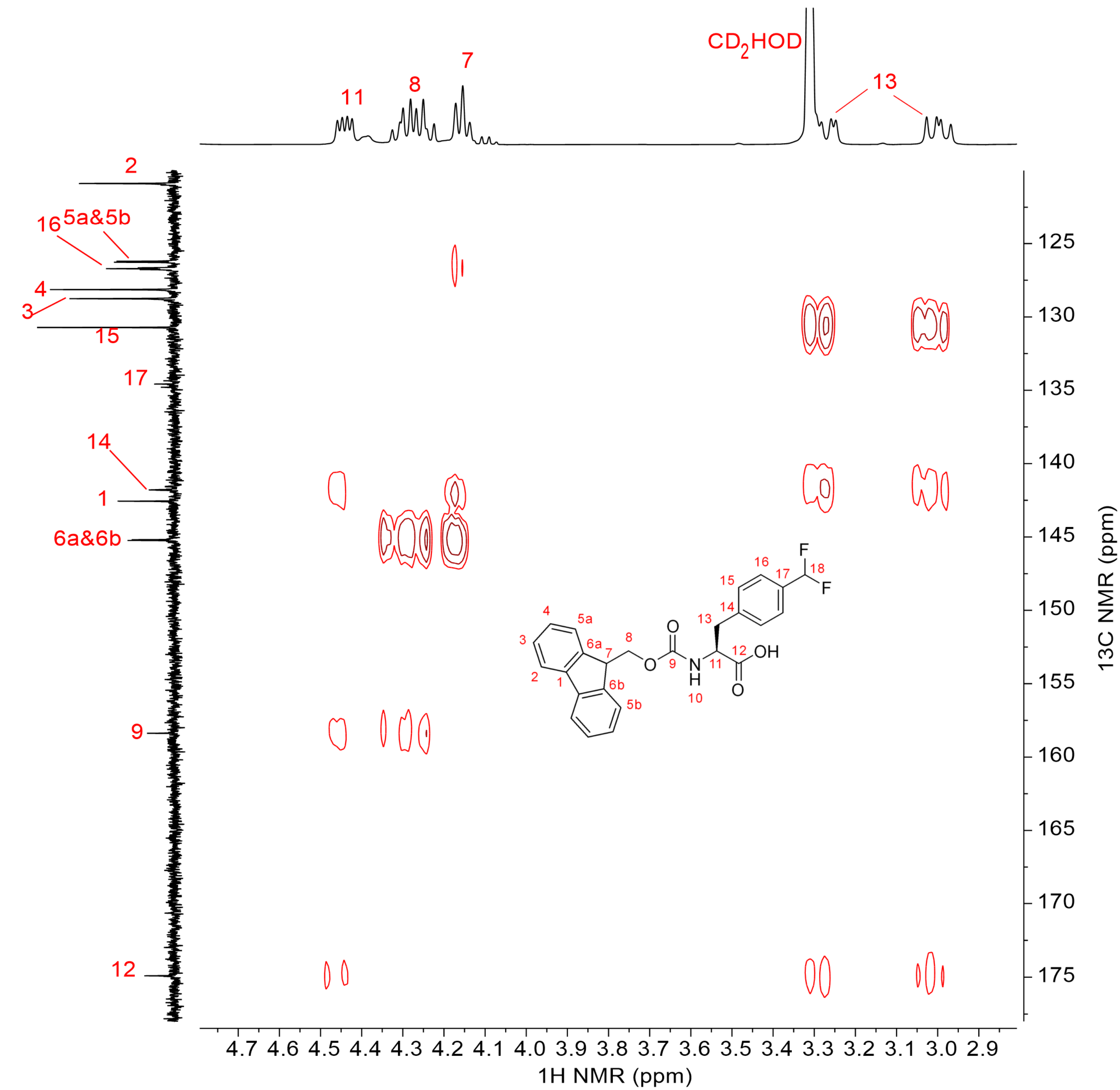

Figure S32. Expansion of ^{1}H-^{13}C HMBC spectrum of *N*-Fmoc-4-difluoromethyl-L-phenylalanine from 4.80 ppm to 2.80 ppm (^{1}H) and 178 ppm to 120 ppm (^{13}C).

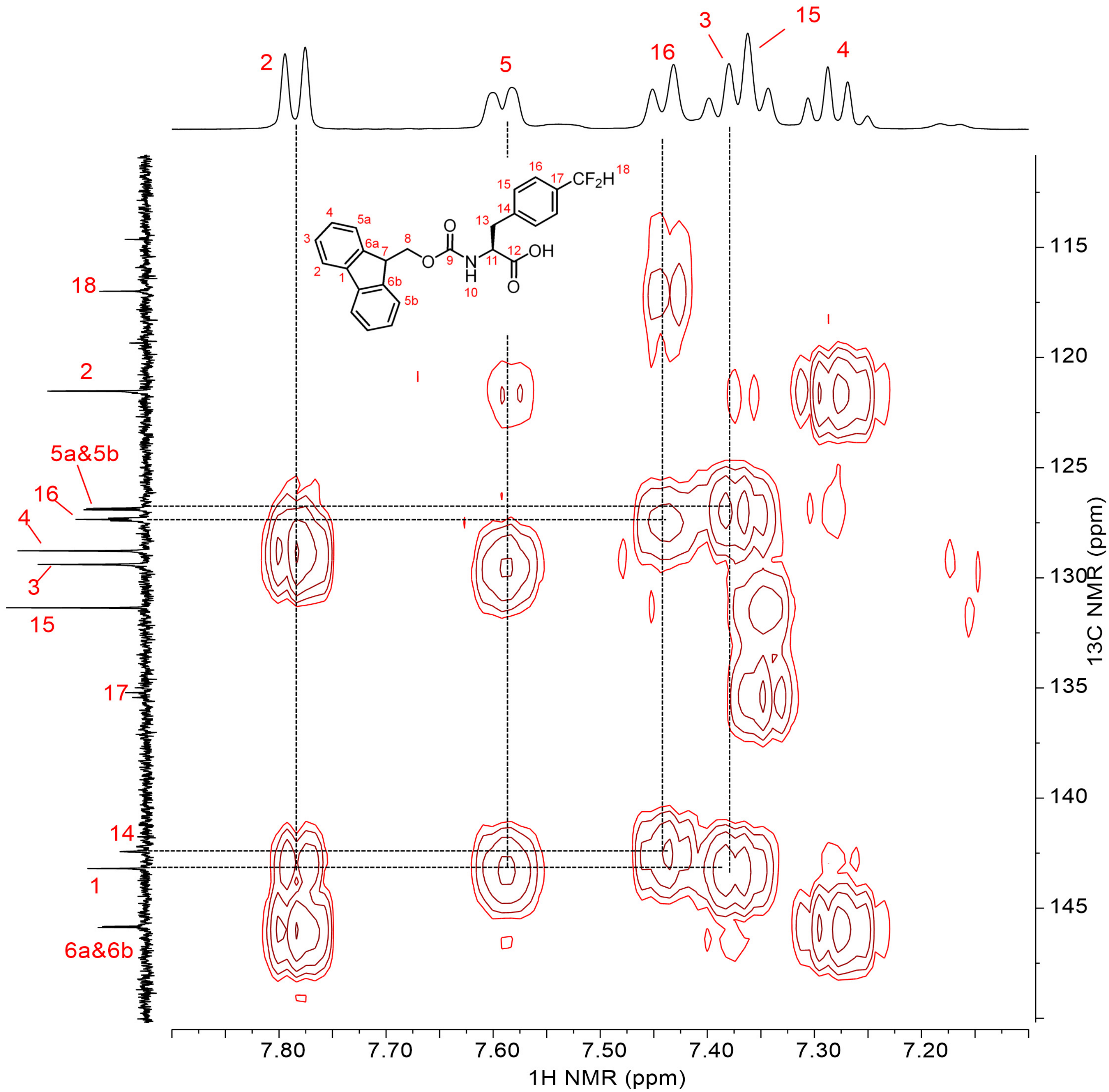

Figure S33. Expansion of ^{1}H-^{13}C HMBC spectrum of *N*-Fmoc-4-difluoromethyl-L-phenylalanine from 7.90 ppm to 7.10 ppm (^{1}H) and 150 ppm to 110 ppm (^{13}C).

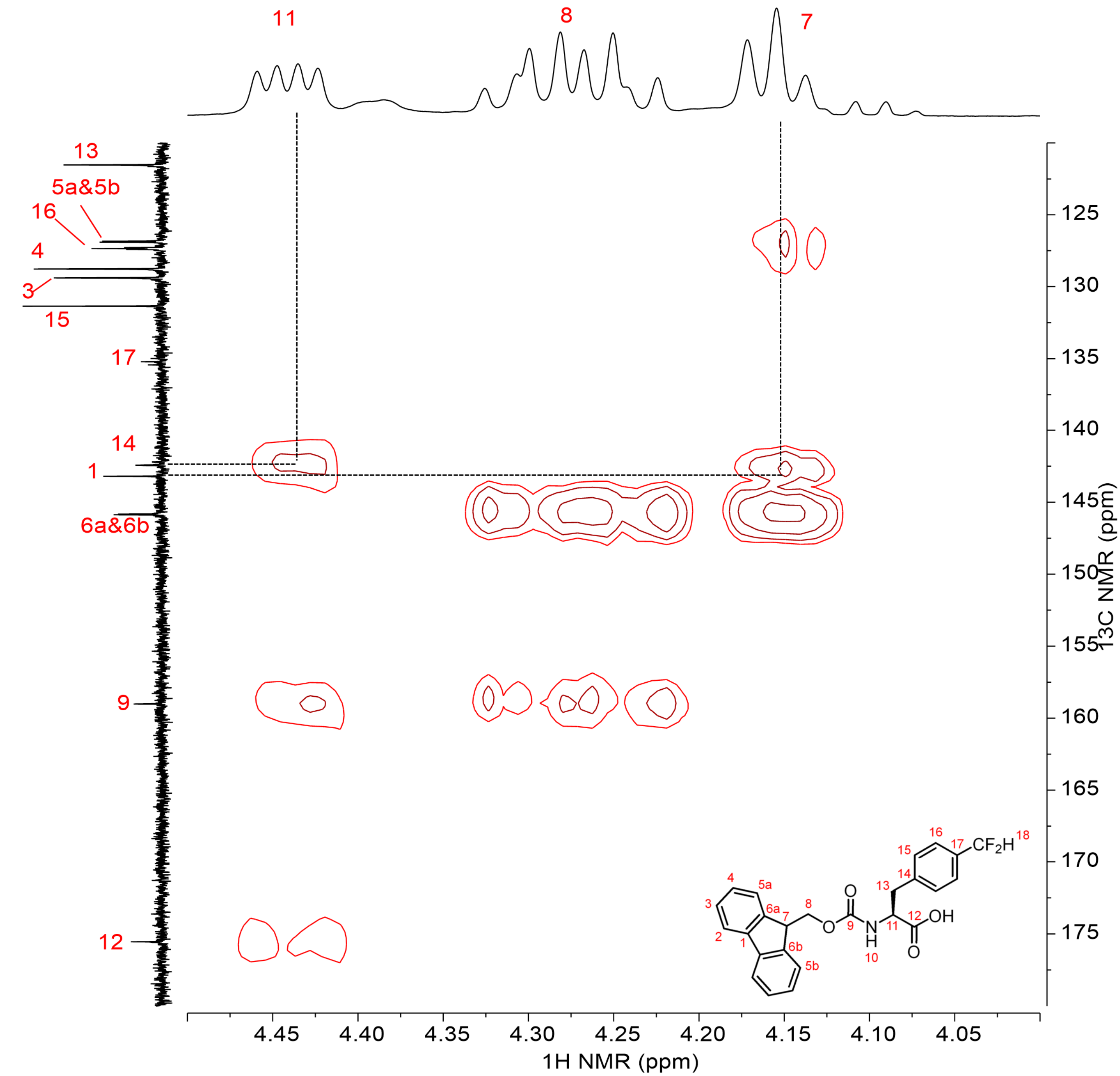

Figure S34. Expansion of ^{1}H-^{13}C HMBC spectrum of *N*-Fmoc-4-difluoromethyl-L-phenylalanine from 4.50 ppm to 4.00 ppm (^{1}H) and 180 ppm to 120 ppm (^{13}C).

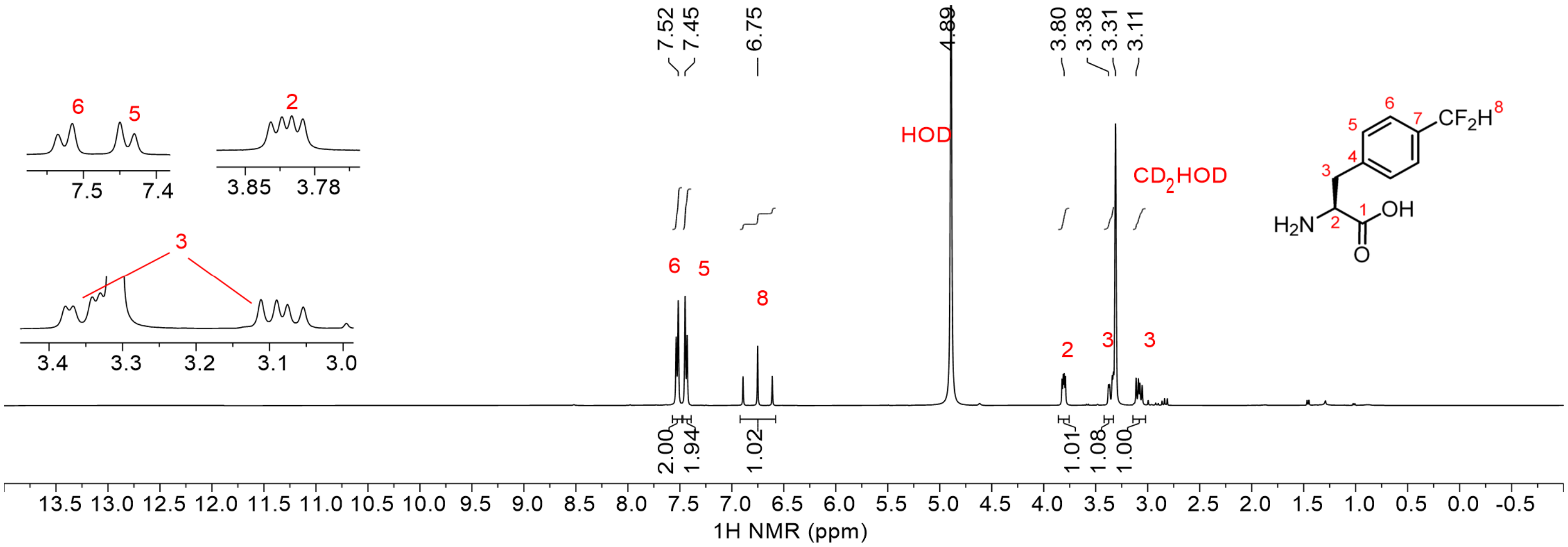

Figure S35. ^{1}H NMR spectrum of 4-difluoromethyl-L-phenylalanine.

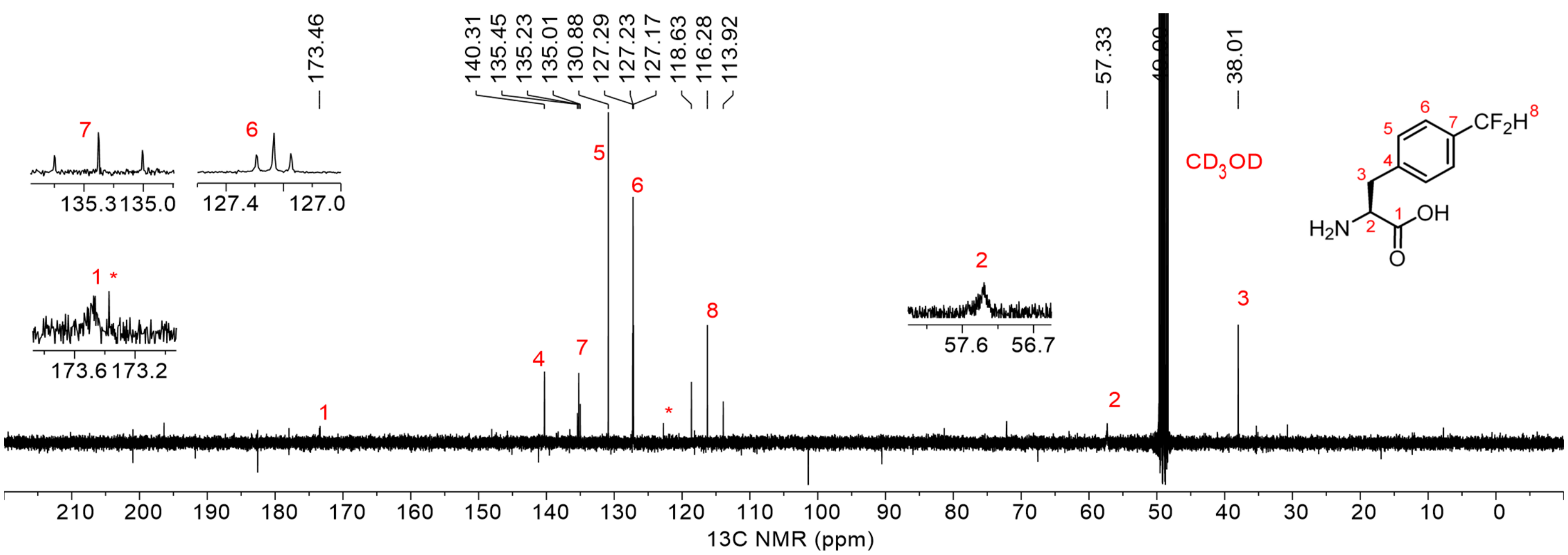

Figure S36. ^{13}C{^{1}H} spectrum of 4-difluoromethyl-L-phenylalanine.

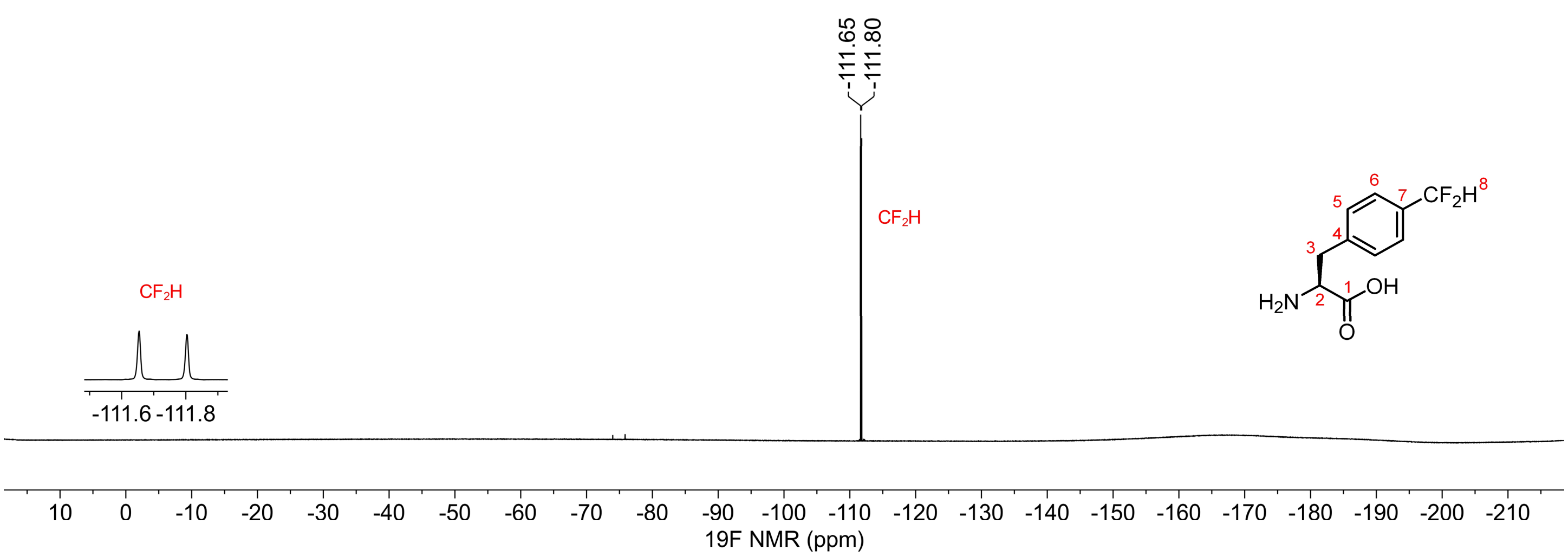

Figure S37. ^{19}F spectrum of 4-Difluoromethyl-L-phenylalanine.

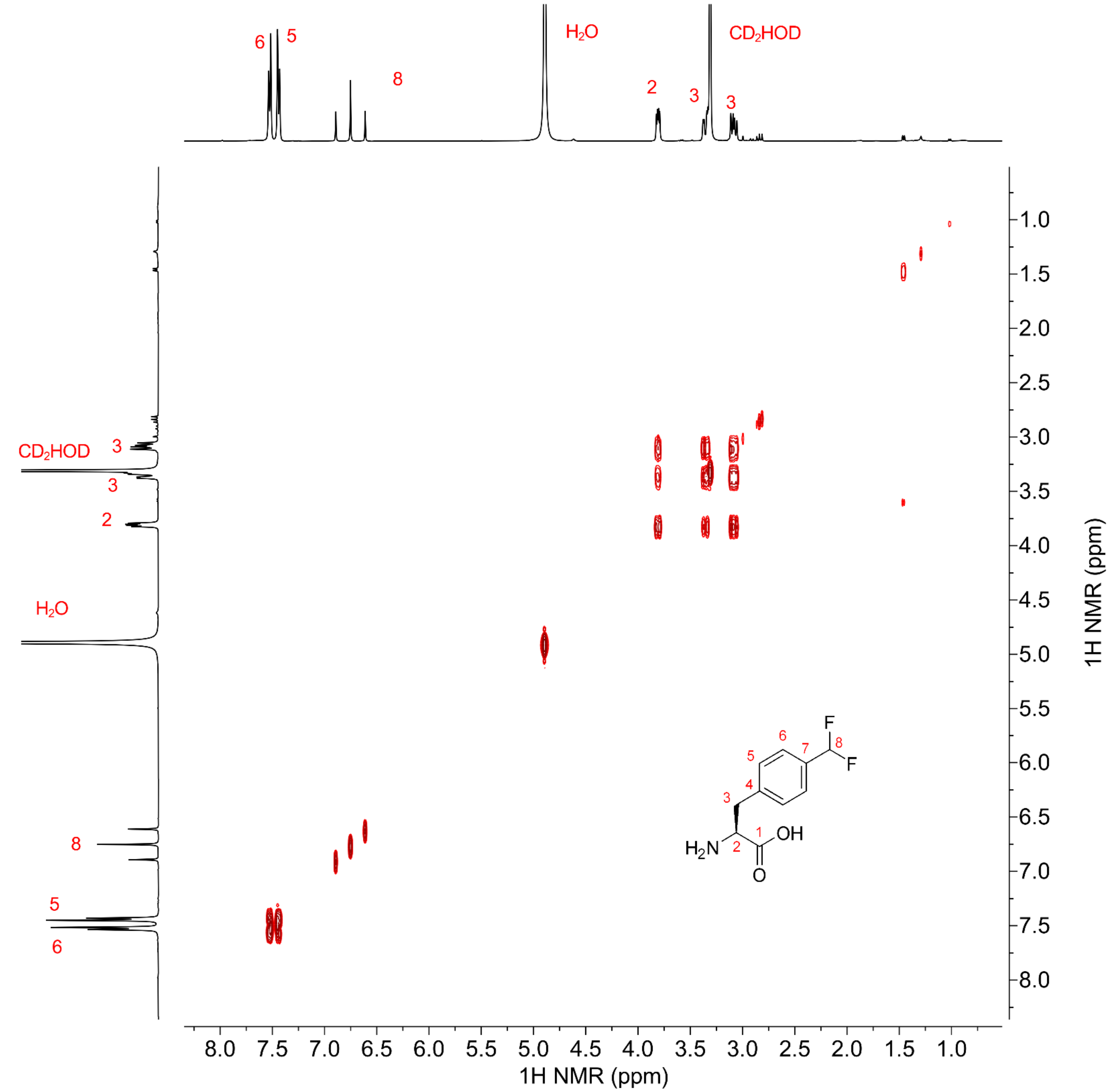

Figure S38. ^{1}H-^{1}H COSY spectrum of 4-difluoromethyl-L-phenylalanine.

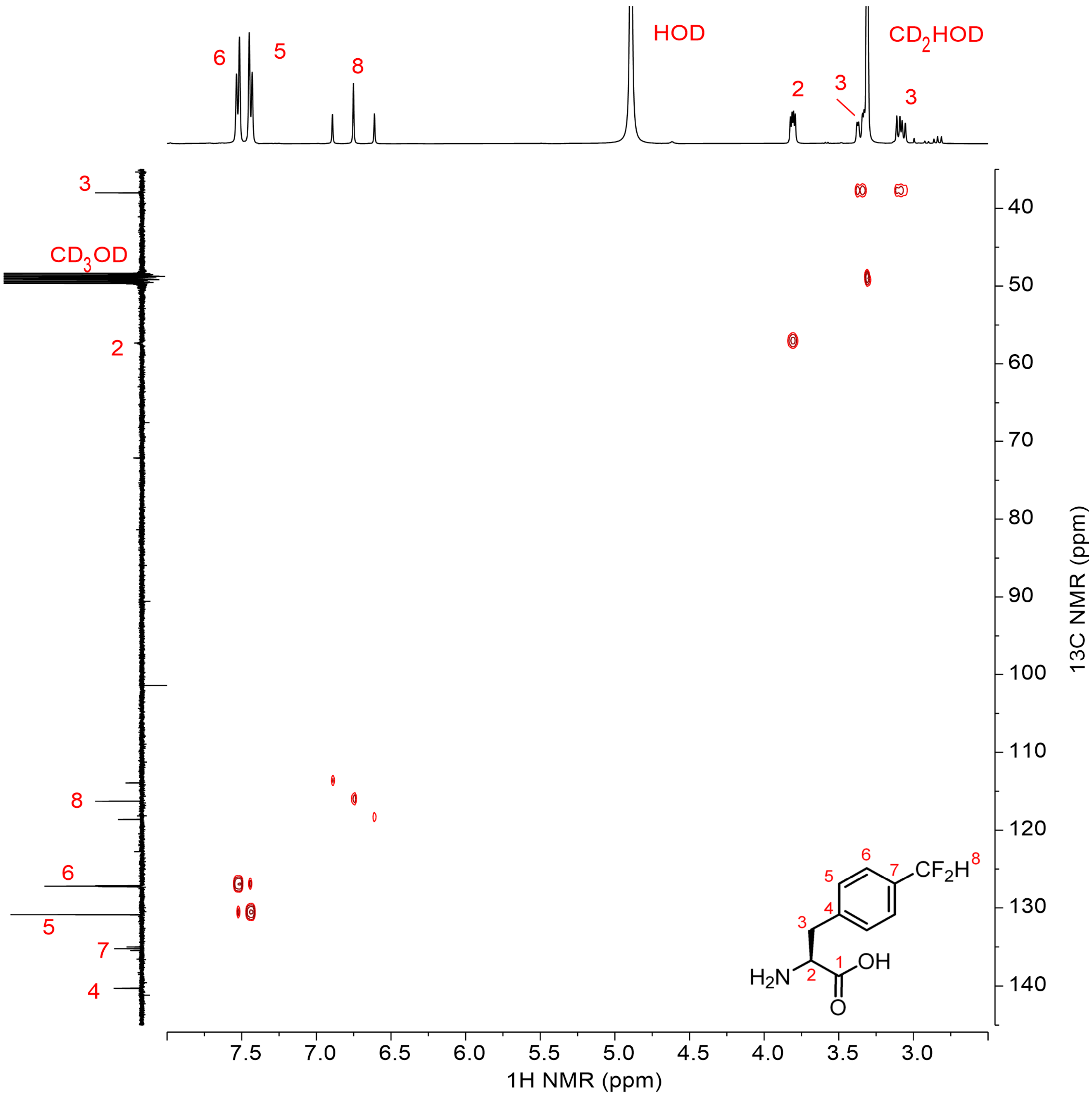

Figure S39. ^{1}H-^{13}C HSQC spectrum of 4-difluoromethyl-L-phenylalanine.

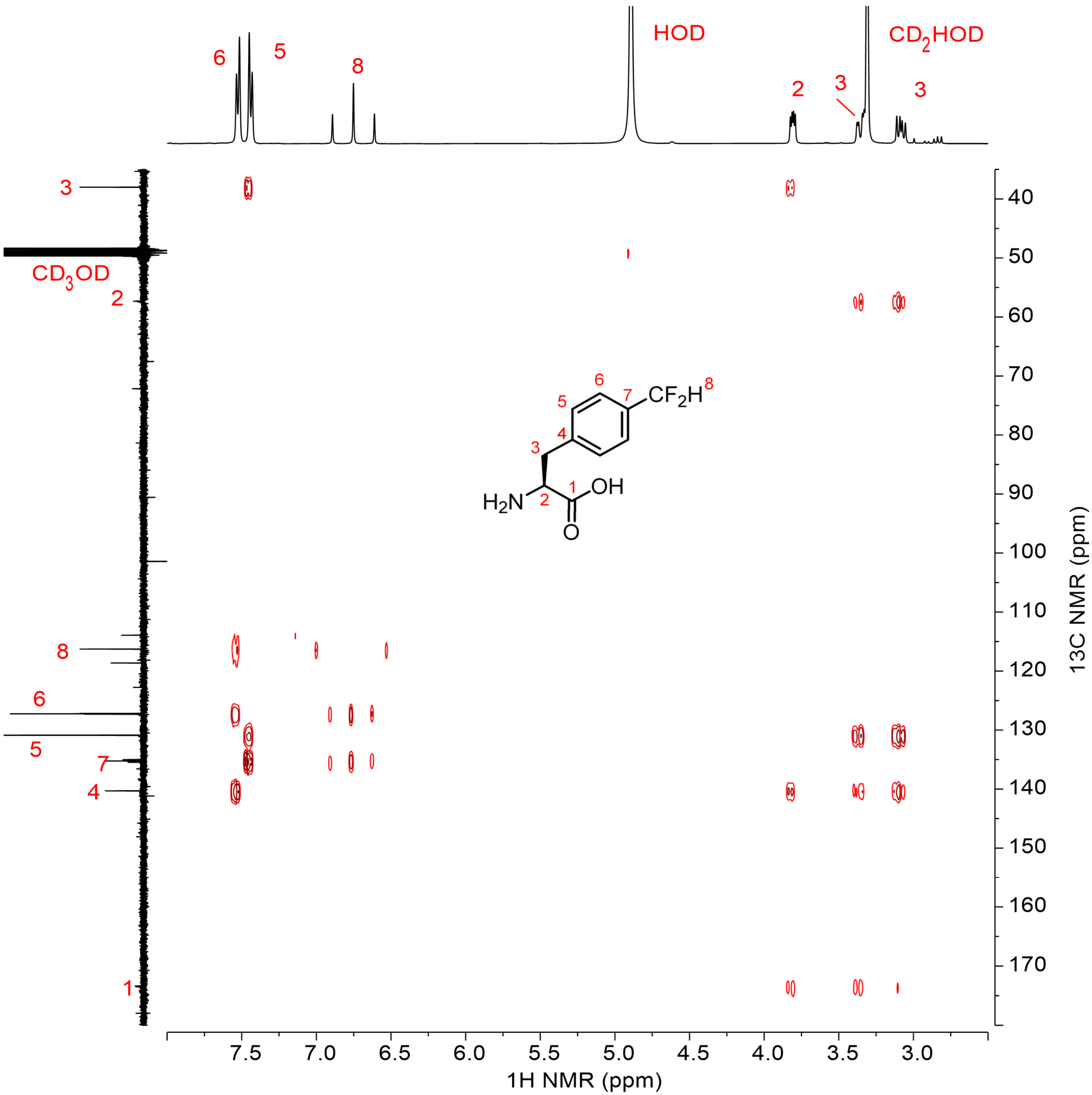

Figure S40. ^{1}H-^{13}C HMBC spectrum of 4-difluoromethyl-L-phenylalanine.

References.

[47] S. Ozturk, C. C. Forneris, A. K. L. Nguy, E. J. Sorensen, and M. R. Seyedsayamdost, "Modulating OxyB-Catalyzed Cross-Coupling Reactions in Vancomycin Biosynthesis by Incorporation of Diverse d-Tyr Analogues," *J. Org. Chem.* **2018**, *83*, 7309-7317.

[54] S. J. Miyake-Stoner, C. A. Refakis, J. T. Hammill, H. Lusic, J. L. Hazen, A. Deiters, and R. A. Mehl, "Generating Permissive Site-Specific Unnatural Aminoacyl-tRNA Synthetases," *Biochemistry* **2010**, *49*, 1667-1677.

[55] J.-D. Pédelacq, S. Cabantous, T. Tran, T. C. Terwilliger, and G. S. Waldo, "Engineering and characterization of a superfolder green fluorescent protein," *Nat. Biotechnol.* **2006**, *24*, 79-88.

[74] J. T. Hammill, S. Miyake-Stoner, J. L. Hazen, J. C. Jackson, and R. A. Mehl, "Preparation of site-specifically labeled fluorinated proteins for ^{19}F-NMR structural characterization," *Nat. Protoc.* **2007**, *2*, 2601-2607.
